# Adamantane-Monoterpenoid Conjugates Linked via Heterocyclic Linkers Enhance the Cytotoxic Effect of Topotecan

**DOI:** 10.3390/molecules27113374

**Published:** 2022-05-24

**Authors:** Aldar A. Munkuev, Nadezhda S. Dyrkheeva, Tatyana E. Kornienko, Ekaterina S. Ilina, Dmitry I. Ivankin, Evgeniy V. Suslov, Dina V. Korchagina, Yuriy V. Gatilov, Alexandra L. Zakharenko, Anastasia A. Malakhova, Jóhannes Reynisson, Konstantin P. Volcho, Nariman F. Salakhutdinov, Olga I. Lavrik

**Affiliations:** 1 N. N. Vorozhtsov Novosibirsk Institute of Organic Chemistry, Siberian Branch of the Russian Academy of Sciences, 9, Akademika Lavrentieva Ave., 630090 Novosibirsk, Russia; amunkuev@nioch.nsc.ru (A.A.M.); ivankind@nioch.nsc.ru (D.I.I.); suslov@nioch.nsc.ru (E.V.S.); korchaga@nioch.nsc.ru (D.V.K.); gatilov@nioch.nsc.ru (Y.V.G.); 2 Institute of Chemical Biology and Fundamental Medicine, Siberian Branch of the Russian Academy of Sciences, 630090 Novosibirsk, Russia; dyrkheeva.n.s@gmail.com (N.S.D.); t.kornienko1995@gmail.com (T.E.K.); katya.plekhanova@gmail.com (E.S.I.); sashaz@niboch.nsc.ru (A.L.Z.); amal@bionet.nsc.ru (A.A.M.); lavrik@niboch.nsc.ru (O.I.L.); 3 Federal Research Centre Institute of Cytology and Genetics, Siberian Branch of the Russian Academy of Sciences, 630090 Novosibirsk, Russia; 4 School of Pharmacy and Bioengineering, Keele University, Hornbeam Building, Newcastle-under-Lyme, Staffordshire ST5 5BG, UK; j.reynisson@keele.ac.uk; 5 Department of Physical and Chemical Biology and Biotechnology, Altai State University, Pr. Lenina 61, 656049 Barnaul, Russia

**Keywords:** tyrosyl-DNA phosphodiesterase 1, adamantane, monoterpene, TDP1 inhibitors, 1,2,4-triazole, 1,3,4-thiadiazole, synergy

## Abstract

Inhibiting tyrosyl-DNA phosphodiesterase 1 (TDP1) is a promising strategy for increasing the effectiveness of existing antitumor therapy since it can remove the DNA lesions caused by anticancer drugs, which form covalent complexes with topoisomerase 1 (TOP1). Here, new adamantane–monoterpene conjugates with a 1,2,4-triazole or 1,3,4-thiadiazole linker core were synthesized, where (+)-and (−)-campholenic and (+)-camphor derivatives were used as monoterpene fragments. The campholenic derivatives **14a**–**14b** and **15a**–**b** showed activity against TDP1 at a low micromolar range with IC_50_ ~5–6 μM, whereas camphor-containing compounds **16** and **17** were ineffective. Surprisingly, all the compounds synthesized demonstrated a clear synergy with topotecan, a TOP1 poison, regardless of their ability to inhibit TDP1. These findings imply that different pathways of enhancing topotecan toxicity other than the inhibition of TDP1 can be realized.

## 1. Introduction

Topoisomerase 1 (TOP1) is a vital DNA repair enzyme participating in the release of torsional tension, resulting from such crucial processes as DNA replication, transcription, chromatin remodeling, and recombination [1,2]. Catalytic mechanism of this enzyme consists in mediating transient DNA single-stranded breaks followed by religation and reestablishment of DNA [3,4]. The inhibition of TOP1 is the basis for treatments with currently used anticancer drugs such as topotecan, irinotecan, and belotecan, which are derivatives of camptothecin, a naturally occurring TOP1 inhibitor [5].

Despite chemotherapy being one of the most common treatments of oncological diseases, there are still many unsolved problems regarding this approach, such as the lack of efficacy and the resistance of tumors. Tyrosyl-DNA phosphodiesterase 1 (TDP1) is believed to be responsible for making cancer cells resistant since this enzyme cleaves the TOP1–DNA covalent complex, thereby repairing DNA damage mediated by TOP1 poisons [6]. Indeed, cells with a higher TDP1 expression have resistance not only to camptothecin, but also to etoposide [7]. Also, TDP1 overexpression prevents the death of colorectal cells when dosed with irinotecan, a frontline chemotherapy used for the treatment of colorectal cancer [8]. Conversely, TDP1 knockout mice were found to be hypersensitive to camptothecin [9,10]. TDP1 depletion in colorectal cancer cells resulted in hypersensitivity to irinotecan. The use of a combination of topotecan and a TDP1 inhibitor led to an increase in the efficacy of the former, both in vitro [11,12,13,14,15] and in vivo [16,17,18]. These findings make TDP1 a legitimate therapeutic target for enhancing the cytotoxic potential of existing anticancer drugs toward malignant cells and overcoming their drug resistance.

**Figure 1:**
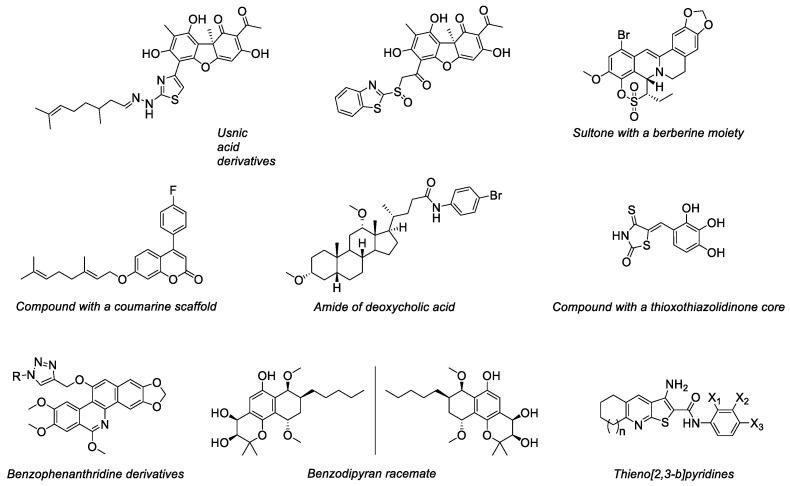
The structural diversity of TDP1 inhibitors.

To date, there are several low-molecular-weight substances exerting anti-TDP1 activity consisting of different organic compounds (Figure 1). These include usnic acid derivatives [19,20], berberine-containing compounds [21], and molecules bearing a coumarine core as a pharmacophore [17]. The scaffold of deoxycholic acid is also a promising platform to develop efficient TDP1 inhibitors [22]. As shown by Sirivolu et al. [23], the use of a thioxothiazolidinone core is a fruitful approach to novel compounds with pronounced inhibitory activity against TDP1. Benzophenanthridine derivatives were demonstrated to be selective TDP1 inhibitors [24]. Curious results were obtained by Wei et.al [25], as they found that a racemic form of benzodipyran with a pyrano [4,3-*h*]chromene scaffold isolated from the soft-coral-derived fungus *Aspergillus* sp. was a more effective TDP1 inhibitor when compared to its optically pure enantiomers. Thieno [2,3-*b*]pyridines also inhibit TDP1 and enhance the cytotoxic effect of the PARP1 (Poly(ADP-Ribose) Polymerase 1) inhibitor olaparib, which indicates the involvement of PARP1 in the repair of this type of DNA damage [26].

**Figure 2:**
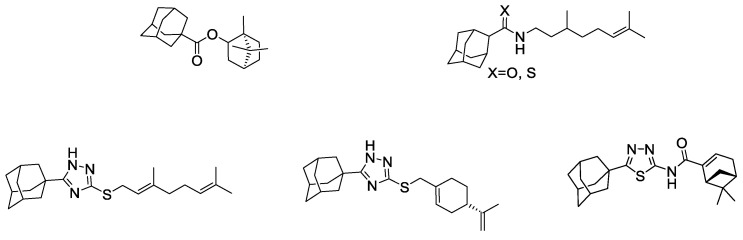
Adamantane derivatives connected with monoterpenoid residues via various linkers as TDP1 inhibitors.

Hybrid molecules consisting of adamantane and monoterpene moieties connected via ester, amide, or thioamide linkers also demonstrate an inhibitory effect on TDP1 [27,28] (Figure 2). In addition, we found that adamantyl-containing 1,2,4-triazoles and 1,3,4-thiadiazoles bearing monoterpenoid moieties not only exerted inhibitory properties against TDP1 in the micromolar/submicromolar range but also enhanced the anticancer cytotoxic effect of topotecan [29].

The results presented here are an extension of our previous work [29] and concentrate on unresolved issues on campholenic and camphor derivatives, thereby complementing existing data on TDP1 inhibitory properties of such hybrid molecules as well as on the ability of such compounds to enhance topotecan activity.

## 2. Results and Discussion

### 2.1. Chemistry

**Scheme 1:**
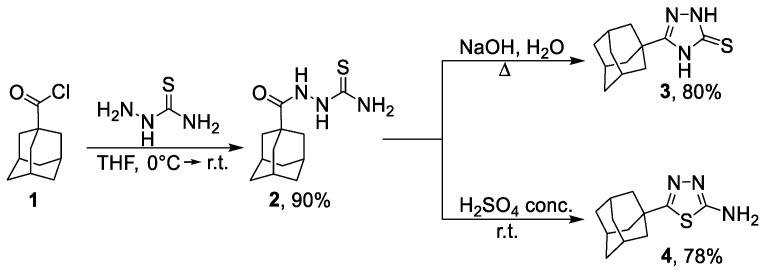
Synthesis of 1,2,4-triazoline **3,** and 1,3,4-thiadiazole **4**.

**Scheme 2:**
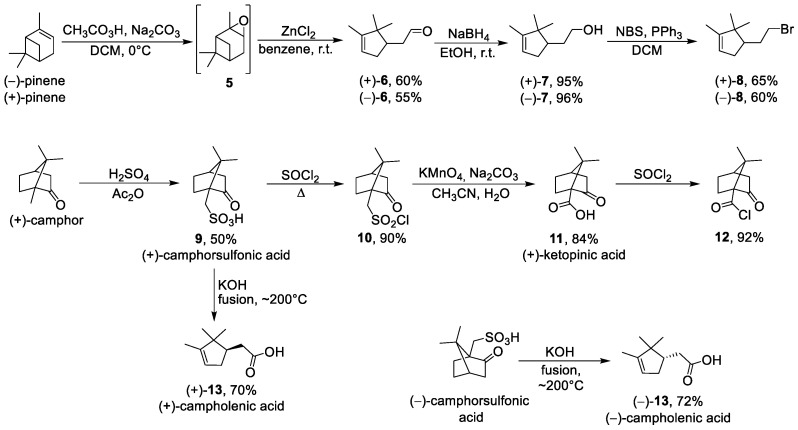
Synthetic route to bromides **8**, campholenic acids **13,** and camphor derivatives **9**, **10**, **11**, **12**.

Firstly, the reaction of acid chloride **1** with thiosemicarbazide gave the corresponding derivative **2** with an excellent 90% yield. The heterocyclic compounds **3** and **4** were obtained by the cyclocondensation of **2** under basic or acidic conditions, respectively (Scheme 1). The structure of amine **4** was confirmed by X-ray crystallography (CCDC 2167259, Supplementary Figure S1).

(+)-Campholenic bromide was prepared using a four-step synthesis starting from (−)-α-pinene as noted in Scheme 2. First, (−)-α-pinene was epoxidized using peracetic acid in DCM to obtain compound **5**, which was used at the next step without further purification. The well-known Meinwald rearrangement of epoxide **5** in the presence of ZnCl_2_ led to the formation of campholenic aldehyde (+)-**6** [30], which was then reduced to campholenic alcohol (+)-**7** using NaBH_4_ in EtOH. The final transformation of the alcohol into corresponding bromide (+)-**8** was carried out using the NBS/PPh_3_ procedure. In order to establish whether there is a difference between enantiomers in TDP1 inhibitory activity, the same reaction sequence was performed to obtain its enantiomer, (−)-campholenic bromide (−)-**8**, starting from (+)-α-pinene.

Reychler’s acid **9** was prepared by the sulfonation of commercially available (+)-camphor with sulfuric acid in acetic anhydride (Scheme 2). The sulfonic acid was then treated with thionyl chloride while refluxing for 2 h to yield sulfonyl chloride **10**. Subsequent oxidation of sulfonyl chloride using KMnO_4_ in water/CH_3_CN mixture at 70 °C for 1.5 h gave (+)-ketopinic acid **11**, in accordance with the procedure reported by Huynh et al. [31]. Thionyl chloride was used to convert ketopinic acid into the corresponding acid chloride **12**. KOH-induced retro-Prince fragmentation of (+)-camphorsulfonic acid **9** under fusion conditions afforded (+)-campholenic acid (+)-**13** [32]. The same reaction sequence with commercial (−)-camphorsulfonic acid was carried out to provide (−)-campholenic acid (−)-**13**.

**Scheme 3:**
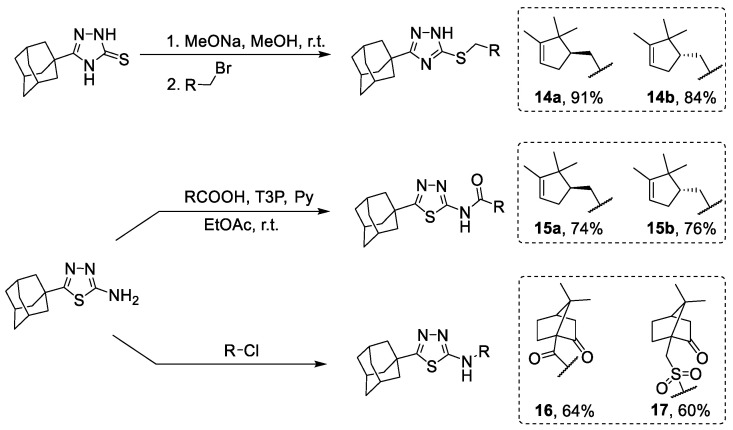
Method of obtaining target derivatives **14a**–**b**, **15a**–**b**, **16**, and **17**.

The reaction of 1,2,4-triazoline **3** with bromides **8** in the presence of MeONa in MeOH allowed us to obtain the desired compounds **14a**-**b** (Scheme 3). (+)- And (−)-campholenic acids **13** were condensed with 1,3,4-thiadiazole **4** in the presence of the mild coupling reagent propanephosphonic acid anhydride (T3P), in order to form amides **15a**–**b**. Sulfonyl chloride **10** and acid chloride **12** were involved in the reaction with amine **4** to give products **16** and **17**.

**Figure 3:**
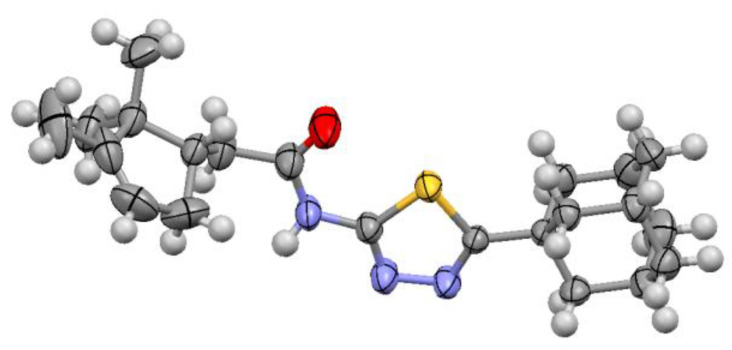
Crystal structure for compound **15b** (50% probability ellipsoids).

Suitable crystals of **15b** (Figure 3) for X-ray diffraction were grown from a hexane–EtOAc mixture, which allowed us to unambiguously confirm the structure of the synthesized campholenic amides (CCDC 2167260, Supplementary Figure S1).

### 2.2. Biology

The compounds were tested against TDP1 using a biosensor assay that was previously developed [33]. The biosensor is a single-stranded oligonucleotide containing a 5′-FAM (5(6)-carboxyfluorescein) fluorophore donor and a quenching 3′-BHQ1 (Black Hole Quencher-1). Since BHQ1 and FAM are located within the Förster radius, the fluorescence is quenched. The mechanism of action is based on the TDP1 enzyme’s ability to cleave substituents selectively from the 3′-end of DNA [34], thus deriving the TDP1 activity by real-time fluorescence detection [33,35]. Furamidine, a commercially available TDP1 inhibitor [35], was used as a positive control; the data are presented in Table 1.

According to Table 1, the campholenic derivatives (**14a**, **14b**, **15a**, **15b**) have similar activity in relation to TDP1 with their IC_50_ value being approximately 5 μM. The camphor derivatives were found to be less active, with compound **16** having a (+)-ketopinic acid fragment and sulfonamide **17** not exhibiting anti-TDP1 activity under experimental conditions.

Next, we tested the compounds’ intrinsic toxicity against cervical cancer HeLa cells and non-cancerous HEK293A immortalized human embryonic kidney cells using the EZ4U assay format. Since TDP1 inhibitors are intended to be used as adjunctive therapy with established chemotherapy drugs, it is important that the compounds do not have inherent side effects. The investigated compounds have moderate toxicity (**14a**, **14b**, **16**) or are non-toxic up to 100 μM (**15a**, **15b**, **17**), (Supplementary Figure S2). The moderate toxicity of conjugates **14a**–**b** is likely due to the presence of a sulfur atom in the side chain, which makes it vulnerable to oxidative processes, whereas the derivatives **15a**–**b**, containing a sulfur atom incorporated in the heterocyclic core, did not show intrinsic cytotoxicity under the experimental conditions.

Then, we tested the ability of the adamantane–monoterpenoid derivatives to sensitize cells to the action of topotecan. For this, we chose a low-toxic concentration 20 μM and varied the concentrations of topotecan (Supplementary Figure S3).

As shown in Table 1 and Figure S3 (Supplementary Figure S3), all the tested compounds exhibited a sensitizing effect on cancer HeLa cells, but the non-cancerous HEK293A cells were unaffected. Thus, the addition of the adamantane–monoterpenoid derivatives led to a decrease by half or more in the semi-toxic concentration (CC_50_) of topotecan against HeLa cells, but not against HEK293A cells. Some compounds such as **14a** and **15b** even protected HEK293A cells from the action of topotecan.

For HeLa cells, we calculated the combination index (CI), the parameter that is used to determine the degree of drug interaction [36]. The combination index (CI) values for the different concentrations (Supplementary Figure S3) of topotecan and TDP1 inhibitors were less than one, and for some compounds less than 0.1 (Supplementary Table S1). This indicates a strong synergistic effect of topotecan and its derivatives. The CI values were calculated with the CompuSyn version 1.0 software.

It should be noted that all the compounds have a synergistic effect on HeLa, regardless of their ability to inhibit TDP1. Obviously, this means that there are additional targets in the cell for these compounds. It is known that TOP1 may have other synthetic lethality partners than TDP1, such as PARP1 [37] or the MPE11 protein [38].

In the reverse experiment, when the concentrations of the compounds were varied with 30 nM topotecan (in the experiments with HEK293A cells) or 3 μM topotecan (in the experiments with HeLa cells), respectively, a noticeable synergistic effect on HeLa cells was already observed at low concentrations of TDP1 inhibitors (1 and 5 μM) (Supplementary Figure S4). Given that the IC_50_ values for these compounds are >5 μM, this also indicates the presence of other targets in addition to TDP1.

In conclusion, the adamantane–monoterpenoid derivatives inhibit TDP1, but apparently have additional cellular targets, since all compounds have a synergistic effect in combination with topotecan, regardless of the effectiveness of TDP1 inhibition, and this effect is manifested at concentrations lower than necessary for TDP1 inhibition.

### 2.3. Molecular Modeling

The six adamantane–monoterpenoid derivatives were docked into the binding site of the TDP1 enzyme (PDB ID: 6W7K, resolution 1.70 Å) [39] using the GOLD molecular modeling software. The robustness of the TDP1 docking scaffold has been previously established [15].

The binding scores for the TDP1 catalytic pocket are given in Table S2 (Supplementary Table S2); all the ligands have reasonable values. No correlation was seen with their corresponding IC_50_ values for any of the scoring functions. Derivatives **16** and **17** have notably higher IC_50_ values than the other ligands but their scores are not statistically different.

**Figure 4:**
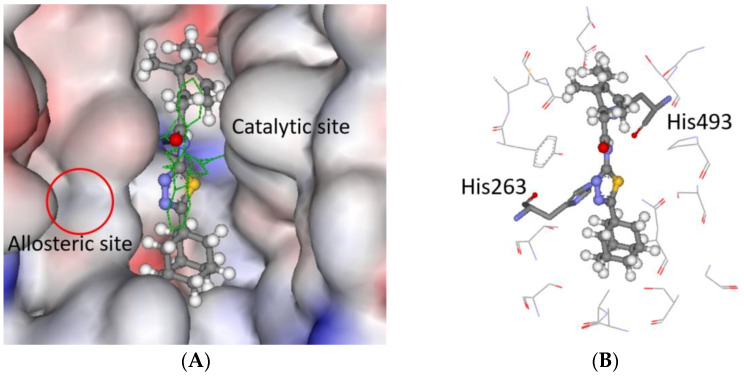
The docked pose of **15b** in the catalytic site of TDP1 as predicted by the ChemPLP scoring function. (**A**) The ligand is shown in the ball-and-stick format. The catalytic pocket is to the right-hand site, the co-crystallised ligand is depicted as green lines. The allosteric pocket is not predicted to be occupied (see circled area). The protein surface is rendered; blue depicts regions with a partial positive charge on the surface; red depicts regions with a partial negative charge; grey shows neutral areas. (**B**) The predicted configuration is depicted as balls-and-sticks, no specific interaction between the ligand and protein was predicted. The catalytic His263 and His493 amino acid residues are shown as sticks. The adjacent amino acids (<5 Å), buttressing the ligand, are shown as lines. The amino acids’ hydrogens are not shown for clarity.

The binding mode of **15b** was investigated since it has good binding to TDP1. For TDP1, all the scoring functions predict the same, or similar, poses, and placed the ligand in the catalytic binding pocket as shown in Figure 4A. The predicted conformation overlaps the co-crystalized ligand and blocks the access to the catalytic site; overall, the ligand fits well into the catalytic pocket. However, no specific interactions such as hydrogen bonding were predicted between the ligand and TDP1 (Figure 4B).

Molecular dynamics simulations have suggested that the TDP1 inhibitors occupy an allosteric binding pocket next to the catalytic site as shown in Figure 4A [40]. The existence of the allosteric site was further supported by combined molecular modeling and a structure–activity relations study of usnic acid derivatives [15]. The occupancy of the allosteric site was shown to be very advantageous to the overall binding efficacy. As can be seen in Figure 4A, the modeling does not predict the occupancy of the allosteric site, the ligand fits well into the catalytic site, and placing either the adamantine or the monoterpenoid into the allosteric site would result in a poor binding configuration. Modifying the central 1,3,4-thiadiazole to carry another monoterpenoid, which could reach into the allosteric site, is an interesting strategy to improve the efficacy of the ligands. Furthermore, having hydrogen bonding interactions in the enzyme would increase the specificity of the ligand, e.g., for ligand **14a,** the triazole ring was predicted by GoldScore to form a hydrogen bond to Ser400. In general, all the ligands were predicted to bind in a similar fashion as **15b**, but in some cases, the adamantine and terpene moieties were inverted.

### 2.4. Chemical Space

The calculated molecular descriptors MW (molecular weight), log P (water-octanol partition coefficient), HD (hydrogen bond donors), HA (hydrogen bond acceptors), PSA (polar surface area), and RB (rotatable bonds) are given in Table S3 (Supplementary Materials). The values of the molecular descriptors lie within a lead-like chemical space for HD and RB; in a lead- and drug-like space for HA and PSA; MW is in a drug-like chemical space; finally, Log P is in a drug-like space and a Known Drug Space (KDS). In general, the ligands are spread over a chemical space depending on the molecular descriptor (for the definition of lead-like, drug-like, and KDS regions see ref. [41] and Table S4 (Supplementary Materials)). When the descriptor values were correlated to their TDP1 IC_50_ counterparts, relatively good R^2^ vales were seen (see Table S3); due to the small number of ligands (*n* = 6), it is doubtful whether a true correlation was observed. Nevertheless, it can be surmised that the binding is governed mainly by water solubility, as the affinity for TDP1 increased with higher log P values (lipophilicity) and lowered with higher HA and PSA values (water solubility), i.e., the entropic term (ΔS) rules this interaction. In addition, according to the modeling, no specific hydrogen bonding interactions were predicted, so the interaction with TDP1 is not driven by enthalpy (ΔH) factors. It is a common view that lipophilicity gives affinity, whereas hydrogen bonding gives specificity, which explains the results from the cell-based assay in which the ligands modulate other targets than TDP1.

The Known Drug Indexes (KDIs) for the ligands were calculated to gauge the balance of the molecular descriptors (MW, log P, HD, HA, PSA, and RB). This method is based on the analysis of drugs in clinical use, i.e., the statistical distribution of each descriptor is fitted to a Gaussian function and normalized to one, resulting in a weighted index. Both the summation of the indexes (KDI_2a_) and multiplication (KDI_2b_) methods were used [42] as shown for KDI_2a_ in Equation (1) and for KDI_2b_ in Equation (2); the numerical results are given in Table S3.

KDI_2a_ = I_MW_ + I_log P_ + I_HD_+ I_HA_ + I_RB_ + I_PSA_ (1)

KDI_2b_ = I_MW_ × I_log P_ × I_HD_× I_HA_ × I_RB_ × I_PSA_ (2)

The KDI_2a_ values for the ligands range from 4.53 to 5.27 with a theoretical maximum of 6 and the average of 4.08 (±1.27) for known drugs. The KDI_2b_ range is from 0.12 to 0.43, with a theoretical maximum of 1 and with KDS average of 0.18 (±0.20). It can be stated that the ligands are reasonably well balanced in terms of their descriptors, making them biocompatible.

## 3. Materials and Methods

### 3.1. Chemistry

All chemicals were purchased from commercial sources (Sigma Aldrich, St. Louis, MO, USA, Acros Organics, Geel, Belgium) and used without further purification. ^1^H and ^13^C NMR spectra were recorded on a Bruker AV-300 spectrometer (300.13 MHz and 75.46 MHz respectively), Bruker AV-400 (400.13 MHz and 100.61 MHz), Bruker DRX-500 (500.13 MHz and 125.76 MHz), and on a Bruker Avance—III 600 (600.30 MHz and 150.95 MHz). Residual signals were used as references (δ_H_ 7.24 and δ_C_ 76.90 ppm for chloroform, δ_H_ 2.50 ppm for DMSO). Compound structures were determined by analyzing the ^1^H-NMR spectra and ^1^H–^1^H 2D homonuclear correlation (COSY, NOESY), J-modulated ^13^C NMR spectra (JMOD), and ^13^C –^1^H 2D heteronuclear correlation with one-bond (HSQC, ^1^J = 145 Hz) and long-range spin–spin coupling constants (HMBC, ^2,3^J = 7 Hz). Mass spectra (70 eV) were recorded on a DFS Thermo Scientific high-resolution mass spectrometer. The conversion of starting materials was analyzed by thin-layer chromatography, which was performed on Merck plates (UV-254). A PolAAr 3005 polarimeter was used to measure optical rotations [α]_D_, where the concentration (c) is shown in g × (100 × mL)^−1^. Target compounds were isolated by column chromatography (SiO_2_; 60–200 µ; Macherey-Nagel). Melting points were measured on a Mettler Toledo FP900 Thermosystem apparatus. Spectral and analytical measurements were carried out at the Multi-Access Chemical Service Center of Siberian Branch of Russian Academy of Sciences (SB RAS). The atom numeration of the substances is provided for the assignment of signals in the NMR spectra and differs from that in IUPAC.

X-ray data for **4** and **15b** compounds X-ray crystallographic data were obtained on a Bruker Kappa Apex II diffractometer with a CCD detector using graphitemonochromated MoKa radiation (λ = 0.71073 Å). Experimental data reduction was performed using the APEX2 suite (Bruker AXS Inc. APEX2 (Version 2.0), SAINT (Version 8.18c), and SADABS (Version 2.11); Bruker Advanced X-ray Solutions, Madison, WI, USA, 2000–2012). The structures were solved by direct methods and refined by the full-matrix least-squares technique against F^2^ in the anisotropic–isotropic approximation. The H atoms positions were calculated with the riding model. All calculations were performed using SHELXTL-2018/3 [43]. CCDC 2167259 (**4**), 2167260 (**15b**) contain the supplementary crystallographic data for this paper. These data can be obtained free of charge from The Cambridge Crystallographic Data Centre via http://www.ccdc.cam.ac.uk/conts/retrieving.html (accessed on 27 April 2022) (or from the CCDC, 12 Union Road, CambridgeCB2 1EZ, UK; Fax: +44-1223-336033; E-mail: deposit@ccdc.cam.ac.uk).

#### 3.1.1. *Synthesis of 2-(Adamantane-1-carbonyl)hydrazine-1-carbothioamide **2***

A solution of adamantane-1-carbonyl chloride (10.0 g, 50.4 mmol) in 30 mL of THF was added to a cooled to 0 °C suspension of thiosemicarbazide (10.08 g, 110.8 mmol) in 200 mL of THF while stirring. The resulting mixture was stirred at room temperature overnight followed by evaporating the solvent on a rotary evaporator. Water was added to the reaction mixture, then the solid mass was filtered off, washed with water thoroughly, and dried. The yield of the compound **2** was 11.48 g (90%), the product was isolated as a white solid.

^1^H-NMR (400 MHz, DMSO-d_6_) (ppm) δ: 1.56–1.72 (m, 6H), 1.75–1.88 (m, 6H), 1.91–2.01 (m, 3H), 6.97 (s, 1H), 7.80 (s, 1H), 9.07 (s, 1H), 9.38 (s, 1H).

#### 3.1.2. *Synthesis of 5-(Adamantan-1-yl)-2,4-dihydro-3H-1,2,4-triazole-3-thione **3***

A mixture containing 5.0 g (8.0 mmol) of 2-(adamantane-1-carbonyl)hydrazine-1-carbothioamide **2** and 1M solution of NaOH (25 mL) was refluxed for 3 h. After cooling to room temperature, the mixture was neutralized using concentrated HCl. The precipitate was collected by filtration, washed with water, and recrystallized from MeOH to give the product as a white solid (1.5 g, 80%).

^1^H-NMR (400 MHz, DMSO-d_6_) (ppm) δ: 1.59–1.71 (m, 6H), 1.80–1.87 (m, 6H), 1.92–1.98 (m, 3H), 13.00 (s, 1H), 13.10 (br.s, 1H).

#### 3.1.3. *Synthesis of 5-(Adamantan-1-yl)-1,3,4-thiadiazol-2-amine **4***

A solution of 2.2 g (8.7 mmol) of 2-(adamantane-1-carbonyl)hydrazine-1-carbothioamide **2** in 20 mL of concentrated sulfuric acid was kept at room temperature overnight and then the solution was neutralized with aqueous ammonia solution while cooling with ice until pH 7 was reached. The resulting solid was filtered off, washed with water, dried, and recrystallized from EtOH to yield 5-(adamantan-1-yl)-1,3,4-thiadiazol-2-amine **4** as a pale yellow solid (1.6 g, 78%).

^1^H-NMR (400 MHz, DMSO-d_6_) (ppm) δ: 1.61–1.76 (m, 6H), 1.84–1.92 (m, 6H), 1.98–2.04 (m, 3H), 6.98 (br.s, 2H).

#### 3.1.4. *Synthesis of (+)-Campholenic Aldehyde **6***

One-round bottom flask was charged with (-)-α-pinene (32.8 mL, 0.207 mol), peracetic acid (32.3 g, 0.248 mol), and sodium carbonate (34.4 g, 0.207 mol) in methylene chloride. The mixture was stirred at 0 °C for 4 h. After filtrating, the solvent was evaporated. The epoxide obtained was dissolved in 600 mL of benzene, ZnCl_2_ (3.0 g) was added, and the mixture was stirred for 16 h at room temperature. The reaction was then quenched with 15 mL of acetic acid and 15 mL of water and the organic phase was separated. The organic phase was again extracted with benzene. The combined organic phase was washed with a saturated solution of sodium bicarbonate and dried over sodium sulfate. After evaporating, the product was purified by silica gel column chromatography to give (+)-campholenic aldehyde **6** as a colorless oil (18.9g, 60%). NMR spectra were consistent with previously reported data [30]. (-)-Campholenic aldehyde was obtained in the same way.

#### 3.1.5. *Synthesis of (+)-Campholenic Alcohol **7***

(+)-Campholenic aldehyde (2.0 g, 13.2 mmol) was dissolved in ethanol (50 mL) and NaBH_4_ was added drop-wise to the solution while stirring. The mixture was stirred at room temperature for 6 h. A saturated solution of NH_4_Cl was then added until gas formation stopped. The product was extracted with Et_2_O and dried over sodium sulfate. Evaporation gave (+)-campholenic alcohol **7** (1.93 g, 95%). NMR spectra were consistent with previously reported data [44]. (-)-Campholenic alcohol was obtained in the same way.

#### 3.1.6. *General Procedure for Synthesis of Bromides **8***

To a solution of PPh_3_ (12.2 g, 46 mmol) in dry DCM (46 mL) was added N-bromosuccinimide (NBS) (8.4 g, 46 mmol) in small portions under an ice-water bath. The mixture was cooled to r.t. and was stirred for 30 min. Then, pyridine (2 mL) was added followed by the corresponding alcohol (24 mmol). The reaction mixture was stirred overnight at room temperature. Then, the mixture was diluted with hexane (60 mL) and filtered through a silica gel plug. The reaction flask was stirred three times with EtOAc:hexane (6:6 mL) for around 1 h and filtered through the silica gel plug. The solution was concentrated in vacuo. The crude residue was purified by flash chromatography (hexane) to obtain corresponding bromide as a colorless oil (3.39 g, 65%).

#### 3.1.7. *(R)-4-(2-Bromoethyl)-1,5,5-trimethylcyclopent-1-ene (+)-**8***


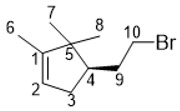
^1^H-NMR (400 MHz, CDCl_3_) (ppm) δ: 0.75 (s, 3H, H-7), 0.97 (s, 3H, H-8), 1.58 (dt, ^4^J = 2.4 Hz, 3H, H-6), 1.75–2.03 (m, 4H, H-3, H-9), 2.23–2.33 (m, 1H, H-4), 3.33 (dt, J = 10 Hz, J = 8 Hz, 1H, H-10a), 3.48 (m, 1H, H-10b), 5.20 (m, 1H, H-2). ^13^C-NMR (100 MHz, CDCl_3_) (ppm) δ: 12.5 (q, C-6), 19.7 (q, C-7), 25.6 (q, C-8), 33.1 (t, C-10), 33.7 (t, C-9), 34.6 (t, C-3), 46.8 (s, C-5), 48.8 (d, C-4), 121.2 (d, C-2), 148.5 (s, C-1). HR MS: 216.0504 (M^+^, C_10_H_16_Br^+^; calc. 216.0508). ${\left[\alpha \right]}_{D}^{25}$ = +24 (c 0.69 in CHCl_3_).

#### 3.1.8. *(S)-4-(2-Bromoethyl)-1,5,5-trimethylcyclopent-1-ene (−)-**8***

NMR spectra were identical to those of (+)-**8**. Colorless oil, yield 60%. ${\left[\alpha \right]}_{D}^{26.5}$ = −25 (c 0.59 in CHCl_3_).

#### 3.1.9. *Synthesis of (+)-10-Camphorsulfonic Acid **9***

An amount of 7.52 mL of concentrated sulfuric acid was added dropwise to 25 mL of acetic anhydride cooled to 0 °C with stirring and the solution was allowed to warm to room temperature. D-camphor (20.0 g) was added to the acid–anhydride solution, and the reaction mixture was kept at room temperature for a month. The reaction mixture was then cooled to a temperature of 0–5 °C and maintained at that temperature for about 6 h. The product was filtered off, washed with Et_2_O, and dried to give 15.4 g (50%) of (+)-camphor-10-sulfonic acid **9** as a white solid. NMR spectra were consistent with previously reported data [45].

#### 3.1.10. *Synthesis of (+)-10-Camphorsulfonyl Chloride **10***

A solution containing 2.0 g of (+)-10-camphorsulfonic acid **9** and 5 mL of thionyl chloride was refluxed for 3 h. After cooling, the solvent was evaporated. The crude product (1.94 g, 90%) was used in the next step without further purification.

#### 3.1.11. *Synthesis of (+)-Ketopinic Acid **11***

A solution of (+)-10-camphorsulfonyl chloride **10** (10.0 g, 39.88 mmol) in MeCN (40 mL) was added to a stirred mixture of Na_2_CO_3_ (12.68 g), KMnO_4_ (13.86 g, 87.74 mmol), H_2_O (140 mL), and MeCN (100 mL). The resulting mixture was stirred for 30 min at r.t., and then heated to 70 °C and stirred for a 1 h. The mixture was allowed to cool to r.t. and then a solution of 3 M H_2_SO_4_ (70 mL) and 2 M Na_2_SO_3_ (164 mL) was added drop-wise. Additional 3 M H_2_SO_4_ was added until the solution turned colorless. The mixture was extracted with Et_2_O, and the combined organic layers were washed with brine, dried over sodium sulfate, and concentrated in vacuo to give (+)-ketopinic acid **11** as an off-white solid (6.12 g, 84%). ^1^H NMR spectra were consistent with previously reported data [31].

#### 3.1.12. *Synthesis of (+)-Ketopinic Acid Chloride **12***

A solution containing 1.55 g (8.5 mmol) of (+)-ketopinic acid, and 1 mL of SOCl_2_ was refluxed for 3 h. The excess of SOCl_2_ was distilled off to give crude material (1.57 g, 92%), which was used directly in the next step.

#### 3.1.13. *Synthesis of (+)-Campholenic Acid **13***

A ground mixture of KOH (1.0 g, 15.68 mmol) and (+)-10-camphorsulfonic acid **9** (1.0 g, 4.3 mmol) was added to a molten KOH (1.0 g). The mixture was stirred with a glass rod for 10 min followed by pouring the mixture into 100 mL of distilled water. The aqueous solution was acidified with concentrated HCl and extracted with Et_2_O. The ether layer was washed with brine and dried over sodium sulfate. The removal of solvents under reduced pressure gave 1.01 g (70%) of (+)-campholenic acid as a yellow oil. ^1^H NMR spectra were consistent with previously reported data [32].

#### 3.1.14. *General Procedure for Obtaining 1,2,4-Triazole Derivatives **14a-b***

To a suspension of compound **3** (0.25 g, 1.06 mmol) in 1 mL of MeOH was added 0.304 mL of 3.5 M solution of MeONa in MeOH. The solution obtained was stirred at r.t. for 30 min, and then corresponding bromide (1 eq.) was added. The reaction mixture was stirred at 60 °C for 12 h and then the solvent was evaporated under reduced pressure. The product was extracted with Et_2_O. The desired compound was purified using column chromatography using a hexane-to-ethyl acetate gradient.

#### 3.1.15. *3-(Adamantan-1-yl)-5-((2-((R)-2,2,3-trimethylcyclopent-3-en-1-yl)ethyl)thio)-1H-1,2,4-triazole **14a***

White solid, mp = 113.5 °C, yield 91%.


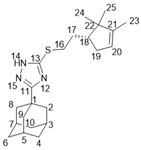
^1^H-NMR (600 MHz, CDCl_3_) (ppm) δ: 0.72 (s, 3H, H-24), 0.93 (s, 3H, H-25), 1.55–1.58 (m, 3H, H-23), 1.59–1.67 (m, 1H, H-17b), 1.70–1.78 (m, 6H, 2H-4, 2H-6, 2H-10), 1.78–1.88 (m, 3H, H-17a, H-18, H-19b), 1.98 (d, ^3^J = 2.9 Hz, 6H, 2H-2, 2H-8, 2H-9), 2.03–2.08 (m, 3H, H-3, H-5, H-7), 2.25–2.34 (m, 1H, H-19a), 3.01 (ddd, J = 12.6, 9.8, 6.4 Hz, 1H, H-16b), 3.16 (ddd, J = 12.6, 10.0, 4.9 Hz, 1H, H-16a), 5.17–5.20 (m, 1H, H-20). ^13^C-NMR (150 MHz, CDCl_3_) (ppm) δ: 34.2 (s, C-1), 40.7 (t, C-2, C-8, C-9), 27.9 (d, C-3, C-5, C-7), 36.3 (t, C-4, C-6, C-10), 166.6 (s, C-11), 159.2 (s, C-13), 31.7 (t, C-16), 30.3 (t, C-17), 49.5 (d, C-18), 35.1 (t, C-19), 121.3 (d, C-20), 148.4 (s, C-21), 46.8 (s, C-22), 12.5 (q, C-23), 19.6 (q, C-24), 25.6 (q, C-25). HR MS: 371.2389 (M^+^, C_22_H_33_N_3_S_1_^+^; calc. 371.2390). ${\left[\alpha \right]}_{D}^{25}$ = +15 (c 0.43 in MeOH).

#### 3.1.16. *3-(Adamantan-1-yl)-5-((2-((S)-2,2,3-trimethylcyclopent-3-en-1-yl)ethyl)thio)-1H-1,2,4-triazole **14b***

NMR spectra were identical to those of **14a**. White solid, mp = 111.8–112.4 °C, yield 84%.

$${\left[\alpha \right]}_{D}^{25}=-17(c\;0.42\;\mathrm{in}\;\mathrm{MeOH}).$$

#### 3.1.17. Synthesis of Amides **15a-b**

To a mixture of campholenic acid (0.9 mmol), amine 4 (1.06 mmol), pyridine (0.264 mL), and EtOAc (0.528 mL) was added propanephosphonic acid anhydride (50 wt% in EtOAc, 2 mmol) while stirring. The reaction mixture was stirred overnight and then water was added. The precipitate formed was washed with water and dried. The product obtained was purified using silica gel column chromatography using a hexane-to-ethyl acetate gradient.

#### 3.1.18. Synthesis of Amides **16–17**

A solution of corresponding carboxylic acid chloride (1.2 mmol) in dry toluene (1 mL) was added to a suspension of amine 17 (1.06 mmol) and triethylamine (0.177 mL, 1.2 mmol) in 10 mL of dry toluene. The reaction mixture was stirred overnight at room temperature and the solvent was evaporated under reduced pressure. The aqueous solution of KOH was added to the residue, followed by extraction with EtOAc. The product was purified using silica gel column chromatography using a hexane-to-ethyl acetate gradient.

#### 3.1.19. N-(5-(Adamantan-1-yl)-1,3,4-thiadiazol-2-yl)-2-((R)-2,2,3-trimethylcyclopent-3-en-1-yl)acetamide **15a**

White solid, mp = 209.2–210.1 °C, yield 74%.


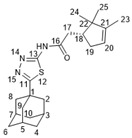
^1^H-NMR (600 MHz, CDCl_3_) (ppm) δ: 0.92 (s, 3H, H-24), 1.04 (s, 3H, H-25), 1.59–1.62 (m, 3H, H-23), 1.72–1.81 (m, 6H, 2H-4, 2H-6, 2H-10), 1.99–2.05 (m, 1H, H-19b), 2.04 (d, ^3^J = 2.8 Hz, 6H, 2H-2, 2H-8, 2H-9), 2.06–2.11 (m, 3H, H-3, H-5, H-7), 2.31–2.42 (m, 2H, H-18, H-19a), 2.77–2.87 (m, 2H, H-17), 5.19–5.22 (m, 1H, H-20), 13.53 (br.s, 1H, NH). ^13^C-NMR (150 MHz, CDCl_3_) (ppm) δ: 37.7 (s, C-1), 43.1 (t, C-2, C-8, C-9), 28.3 (d, C-3, C-5, C-7), 36.3 (t, C-4, C-6, C-10), 174.4 (s, C-11), 159.8 (s, C-13), 172.1 (s, C-16), 37.1 (t, C-17), 46.7 (d, C-18), 35.3 (t, C-19), 121.6 (d, C-20), 147.8 (s, C-21), 47.0 (s, C-22), 12.6 (q, C-23), 20.0 (q, C-24), 25.5 (q, C-25). HR MS: 385.2185 (M^+^, C_22_H_31_O_1_N_3_S_1_^+^; calc. 385.2182). ${\left[\alpha \right]}_{D}^{24.5}$ = +49 (c 0.41 in CHCl_3_).

#### 3.1.20. N-(5-(Adamantan-1-yl)-1,3,4-thiadiazol-2-yl)-2-((S)-2,2,3-trimethylcyclopent-3-en-1-yl)acetamide **15b**

White solid, mp = 210.9 °C, yield 76%.

NMR spectra were identical to those of **15a**. ${\left[\alpha \right]}_{D}^{24.5}$ = −45 (c 0.41 in CHCl_3_).

#### 3.1.21. (1S,4R)-N-(5-(Adamantan-1-yl)-1,3,4-thiadiazol-2-yl)-7,7-dimethyl-2-oxobicyclo[2.2.1]heptane-1-carboxamide **16**

White solid, mp = 269.6 °C, yield 64%.


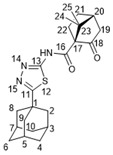
^1^H-NMR (600 MHz, CDCl_3_) (ppm) δ: 1.00 (s, 3H, H-24), 1.28 (s, 3H, H-25), 1.49 (ddd, J = 12.8, 9.2, 4.0 Hz, 1H, H-21endo), 1.70–1.82 (m, 7H, 2H-4, 2H-6, 2H-10, H-22b), 2.02–2.11 (m, 10H, H-3, H-5, H-7, 2H-2, 2H-8, 2H-9, H-19endo), 2.12–2.16 (m, 1H, H-20), 2.15–2.23 (m, 1H, H-21exo), 2.46–2.53 (m, 1H, H-22endo), 2.59 (dm, ^2^J=18.8 Hz, 1H, H-19exo), 11.04 (br.s, 1H, NH). ^13^C-NMR (150 MHz, CDCl_3_) (ppm) δ: 37.6 (s, C-1), 43.1 (t, C-2, C-8, C-9), 28.3 (d, C-3, C-5, C-7), 36.3 (t, C-4, C-6, C-10), 175.4 (s, C-11), 156.6 (s, C-13), 167.3 (s, C-16), 64.1 (s, C-17), 215.5 (s, C-18), 43.4 (t, C-19), 43.3 (d, C-20), 27.7 (t, C-21), 29.1 (t, C-22), 50.7 (s, C-23), 20.2 (q, C-24), 20.6 (q, C-25). HR MS: 399.1973 (M^+^, C_22_H_29_O_2_N_3_S_1_^+^; calc. 399.1975). ${\left[\alpha \right]}_{D}^{26.5}$ = +16 (c 0.47 in CHCl_3_).

#### 3.1.22. N-(5-(Adamantan-1-yl)-1,3,4-thiadiazol-2-yl)-1-((1S,4R)-7,7-dimethyl-2-oxobicyclo[2.2.1]heptan-1-yl)methanesulfonamide **17**

White solid, mp = 200.3–200.5 °C, yield 60%.


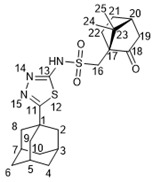
^1^H-NMR (600 MHz, CDCl_3_) (ppm) δ: 0.83 (s, 3H, H-24), 1.09 (s, 3H, H-25), 1.41 (ddd, J = 12.7, 9.4, 3.5 Hz, 1H, H-21endo), 1.66–1.79 (m, 7H, 2H-4, 2H-6, 2H-10, H-22b), 1.90–1.96 (m, 7H, 2H-2, 2H-8, 2H-9, H-19endo), 1.98–2.05 (m, 1H, H-21exo), 2.05–2.10 (m, 4H, H-3, H-5, H-7, H-20), 2.32 (ddd, J = 18.5, 4.8, 3.5 Hz, 1H, H-19exo), 2.55 (ddd, J = 14.5, 11.7, 4.0 Hz, 1H, H-22a), 3.00 (d, J = 14.8 Hz, 1H, H-16b), 3.56 (d, J = 14.8 Hz, 1H, H-16a). ^13^C-NMR (150 MHz, CDCl_3_) (ppm) δ: 38.2 (s, C-1), 41.9 (t, C-2, C-8, C-9), 28.0 (d, C-3, C-5, C-7), 36.1 (t, C-4, C-6, C-10), 168.1 (s, C-11), 167.9 (s, C-13), 49.8 (t, C-16), 58.2 (s, C-17), 215.7 (s, C-18), 42.5 (t, C-19), 42.5 (d, C-20), 26.8 (t, C-21), 24.4 (t, C-22), 48.1 (s, C-23), 19.6 (q, C-24), 19.8 (q, C-25). HR MS: 449.1803 (M^+^, C_22_H_31_O_3_N_3_S_2_^+^; calc. 449.1801). ${\left[\alpha \right]}_{D}^{26.5}$ = +29 (c 0.47 in CHCl_3_).

### 3.2. Biological Assays

#### 3.2.1. Detection of TDP1 Activity

The methodology has been reported in our previous work [33] and consists of fluorescence intensity measurements in a reaction of quencher removal from a fluorophore quencher-coupled DNA oligonucleotide catalyzed by TDP1. The reaction was carried out at different concentrations of inhibitors (the control samples contained 1% of DMSO, Sigma, St. Louis, MO, USA). The reaction mixtures contained TDP1 buffer (50 mM Tris-HCl pH 8.0, 50 mM NaCl, and 7 mM β-mercaptoethanol), 50 nM biosensor, and the inhibitor being tested. Purified TDP1 (1.5 nM) triggered the reaction. The biosensor (5′-[FAM] AAC GTC AGGGTC TTC C [BHQ]-3′) was synthesized in the Laboratory of Nucleic Acids Chemistry at the Institute of Chemical Biology and Fundamental Medicine (Novosibirsk, Russia).

The reactions were incubated on a POLARstar OPTIMA fluorimeter (BMG LABTECH, GmbH, Ortenberg, Germany) to measure the fluorescence every 60 s (ex. 485/em. 520 nm) during the linear phase (here, data from minute 0 to minute 8). The values of IC_50_ were determined using a six-point concentration response curve in a minimum of three independent experiments and were calculated using MARS Data Analysis 2.0 (BMG LABTECH, GmbH, Ortenberg, Germany).

#### 3.2.2. Cytotoxicity Assays

The cytotoxicity of the compounds to HeLa (human cervical cancer) and HEK293A (human embryonic kidney) cell lines was examined using the EZ4U Cell Proliferation and Cytotoxicity Assay (Biomedica, Vienna, Austria), according to the manufacturer’s protocols. The cells were grown in DMEM (Dulbecco’s Modified Eagle’s Medium) with 50 IU/mL penicillin, 50 μg/mL streptomycin (MP Biomedicals, Santa Ana, CA, USA), and 10% of fetal bovine serum (Biolot, St. Petersburg, Russia) in a 5% CO_2_ atmosphere. After formation of a 30–50%-monolayer, the tested compounds were added to the medium. The volume of the added reagents was 1/100 of the total volume of the culture medium, and the amount of DMSO (Sigma, St. Louis, MO, USA) was 1% of the final volume. The cell culture was monitored for 3 days. To assess the influence of the inhibitors on the cytotoxic effect of topotecan (Selleck Chemicals, Houston, TX, USA), 50% cytotoxic concentrations of topotecan and of each inhibitor were determined to attain a defined single-agent effect. Then, a minimum of two independent tests were performed with each inhibitor in combination with topotecan. When using a combination of drugs, TDP1 inhibitors were first added, then topotecan was added immediately (within 10–15 min).

### 3.3. Modeling

The compounds were docked against the crystal structure of TDP1 (PDB ID: 6W7K, resolution 1.70 Å) [39], which was obtained from the Protein Data Bank (PDB) [46,47]. The GOLD (v2020.2.0) software suite was used to prepare the crystal structures for docking, i.e., the hydrogen atoms were added, water molecules deleted, and the co-crystallised ligands were identified: Tdp1-4-[(2-phenylimidazo[1,2-a]pyridin-3-yl)amino]benzene-1,2-dicarboxylic acid (TG7). The Scigress version FJ 2.6 program [48] software suite was used to build the ligands and the MM3 [49,50,51] force field was applied to identify the global minimum using the CONFLEX method [52], followed by structural optimisation. The docking centre for the TDP1 catalytic pocket was defined as the position of the co-crystalised ligand TG7. Fifty docking runs were allowed for each ligand with default search efficiency (100%). The basic amino acids lysine and arginine were defined as being protonated. Furthermore, aspartic and glutamic acids were assumed to be deprotonated. The GoldScore(GS) [53] and ChemScore(CS) [54,55] ChemPLP(Piecewise Linear Potential) [56] and ASP(Astex Statistical Potential) [57] scoring functions were implemented to predict the binding modes and relative binding energies of the ligands using the GOLD v2020.2.0 software suite.

The QikProp 6.2 [58] software package was used to calculate the molecular descriptors of the molecules. The reliability of the QikProp was established for the calculated descriptors [59]. The Known Drug Indexes (KDI) were calculated from the molecular descriptors as described by Eurtivong and Reynisson [42]. For application in Excel, columns for each property were created and the following equations used do derive the KDI numbers for each descriptor: KDI MW: = EXP(-((MW-371.76)^2^)/(2 × (112.76^2^))), KDI Log P: = EXP(-((LogP-2.82)^2^)/(2 × (2.21^2^))), KDI HD: = EXP(-((HD-1.88)^2^)/(2 × (1.7^2^))), KDI HA: = EXP(-((HA-5.72)^2^)/(2 × (2.86^2^))), KDI RB = EXP(-((RB-4.44)^2^)/(2 × (3.55^2^))), and KDI PSA: = EXP(-((PSA-79.4)^2^)/(2 × (54.16^2^))). These equations could simply be copied into Excel, with the descriptor name (e.g., MW) substituted being the value in the relevant column. To derive KDI_2A_, this equation was used: = (KDI MW + KDI LogP + KDI HD + KDI HA + KDI RB + KDI PSA) and for KDI_2B_: = (KDI MW × KDI LogP × KDI HD × KDI HA × KDI RB × KDI PSA).

## 4. Conclusions

For the first time, adamantane–monoterpene conjugates with a 1,2,4-triazole or 1,3,4-thiadiazole nucleus were synthesized, where campholenic or camphor moieties were used as a monoterpene part. The inhibitory activity of the compounds bearing campholenic fragments were shown to be in a narrow IC_50_ range of ~5 μM regardless of the heterocyclic core or the configuration of the stereocenter in the monoterpene moiety. The inhibitory activity of these molecules is in accordance with the previously reported data [29], where structurally similar compounds were studied. Interestingly, camphor derivatives were found to be ineffective inhibitors of TDP1. Apparently, the presence of a keto-group in the monoterpene scaffold has a negative effect on TDP1 inhibition, but not on the ability to potentiate topotecan, a TOP1 poison, since all the compounds tested have a synergistic effect, which is, at least partly, due to the presence of other mechanisms that enhance topotecan cytotoxicity towards cancer cells.

## Figures and Tables

**Table 1:** Data on TDP1 inhibition (IC_50_) as well as cytotoxicity (CC_50_) on HeLa and HEK293A cell lines. Furthermore, the influence on topotecan (**Tpc)** cytotoxicity was measured.

	Structure	TDP1 IC_50_, μM	HeLa CC_50_, μM	HEK293A CC_50_, μM	Tpc CC_50_ HeLa, µM	Tpc CC_50_ HEK293A, nM
**Tpc**					4.0 ± 1.3	66 ± 28
**14a**	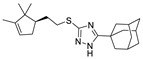	5.5 ± 1.5	53 ± 8	47 ± 3	0.97 ± 0.23	240 ± 60
**14b**	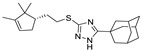	6.1 ± 0.1	54 ± 7	50 ± 3	1.2 ± 0.4	63 ± 23
**15a**	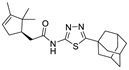	6.1 ± 2.6	>100	>100	1.7 ± 0.6	79 ± 20
**15b**	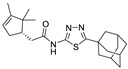	5.6 + 1.9	>100	>100	2.2 ± 0.8	140 ± 45
**16**	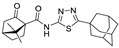	>30	20 ± 8	23 ± 10	1.6 ± 0.4	120 ± 33
**17**	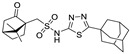	>30	100 ± 20	>100	1.9 ± 0.7	95 ± 25
Furamidine		1.2 ± 0.3				

## Data Availability

The data presented in this study are available on request from the corresponding author.

## Supplementary Materials

The following are available online: https://www.mdpi.com/article/10.3390/molecules27113374/s1. Figure S1: Crystal structure for compounds **4** and **15b**; Table S1: The combination index (CI) values for different concentrations of topotecan and TDP1 inhibitors; Figure S2: The cytotoxicity of the monoterpene derivatives against HeLa and HEK293A cells; Figure S3: The influence of the TDP1 inhibitors on topotecan cytotoxicity derivatives against HeLa and HEK293A cells; Figure S4: The influence of topotecan on the adamantane–monoterpenoid derivatives’ cytotoxicity; Table S2: The binding affinities as predicted by the scoring functions used to the catalytic TDP1 binding pocket; Table S3: The molecular descriptors and their corresponding Known Drug Indexes 2a and 2b (KDI2a/2b); Table S4: Definition of lead-like, drug-like, and Known Drug Space (KDS) in terms of molecular descriptors; Figures S5–S29: ^1^H, ^13^C NMR, DFS, and IR spectra of compounds **8**, **14a**–**14b**, **15a**–**b**, **16** and **17**.

## Sample Availability

Not available.
