# Supplementary material for: Adamantane-Monoterpenoid Conjugates Linked via Heterocyclic Linkers Enhance the Cytotoxic Effect of Topotecan
Source: Molecules. 2022 May 24;27(11):3374. doi: 10.3390/molecules27113374 (PMC9182348; doi:10.3390/molecules27113374)
Supplement: Supplementary file 1 [file molecules-27-03374-s001.zip › molecules-1726599-supplementary.pdf]

Adamantane-monoterpenoid conjugates linked via heterocyclic linkers enhance cytotoxic effect of topotecan

Aldar A. Munkuev[1], Nadezhda S. Dyrkheeva[2], Tatyana E. Kornienko,[2] Ekaterina S. Ilina[2], Dmitry I. Ivankin[1], Evgeniy V. Suslov[1], Dina V. Korchagina[1], Yuriy V. Gatilov[1], Alexandra L. Zakharenko[2], Anastasia A. Malakhova[2,4], Jóhannes Reynisson[3], Konstantin P. Volcho[1*], Nariman F. Salakhutdinov[1*], Olga I. Lavrik[2,5]

1 N. N. Vorozhtsov Novosibirsk Institute of Organic Chemistry, Siberian Branch of the Russian Academy of Sciences, 9, Akademika Lavrentieva Ave., 630090 Novosibirsk, Russia; amunkuev@nioch.nsc.ru (A.A.M.); ivankind@nioch.nsc.ru (D.I.I.); suslov@nioch.nsc.ru (E.V.S.); korchaga@nioch.nsc.ru (D.V.K.); gatilov@nioch.nsc.ru (Y.V.G.), volcho@nioch.nsc.ru (K.P.V.); anvar@nioch.nsc.ru (N.F.S.)
2 Institute of Chemical Biology and Fundamental Medicine, Siberian Branch of the Russian Academy of Sciences, Novosibirsk 630090, Russian Federation; dyrkheeva.n.s@gmail.com (N.S.D.); t.kornienko1995@gmail.com (T.E.K.); sashaz@niboch.nsc.ru (A.L.Z.); katya.plekhanova@gmail.com (E.S.I.); lavrik@niboch.nsc.ru (O.I.L.)
3 School of Pharmacy and Bioengineering, Keele University, Hornbeam Building, Staffordshire ST5 5BG, UK; j.reynisson@keele.ac.uk
4 Federal Research Centre Institute of Cytology and Genetics, Siberian Branch of the Russian Academy of Sciences, 630090 Novosibirsk, Russia
5 Department of Physical and Chemical Biology and Biotechnology, Altai State University, Pr. Lenina 61, 656049 Barnaul, Russia
* Correspondence: volcho@nioch.nsc.ru; anvar@nioch.nsc.ru

Supplementary materials

Table of contents

Crystal structure for compounds **4** and **15b**	**Figure S1**
The combination index (CI) values for different concentrations of topotecan and Tdp1 inhibitors	**Table S1**
The cytotoxicity of the monoterpene derivatives against HeLa and HEK293A cells	**Figure S2**
The influence of the TDP1 inhibitors on topotecan cytotoxicity derivatives against HeLa and HEK293A cells	**Figure S3**
The influence of topotecan on the adamantane-monoterpenoid derivatives' cytotoxicity	**Figure S4**
The binding affinities as predicted by the scoring functions used to the catalytic Tdp1 binding pocket	**Table S2**
The molecular descriptors and their corresponding Known Drug Indexes 2a and 2b ($KDI_{2a/2b}$)	**Table S3**
Definition of lead-like, drug-like and Known Drug Space (KDS) in terms of molecular descriptors	**Table S4**
^{1}H, ^{13}C NMR, DFS and IR spectra of compounds **8**, **14a-14b**, **15a-b**, **16** and **17**	**Figures S5-S29**

Chemical structures of the molecules **4** and **15b** obtained by X-ray single crystal diffractometry:

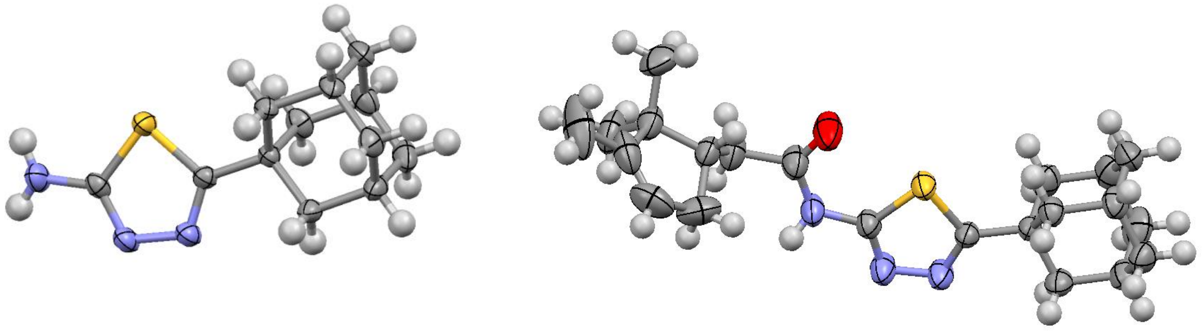

4 **15b**

Figure S1. Crystal structure for compounds **4** and **15b** (50% probability ellipsoids)

Crystal data **4**: $C_{12}H_{17}N_3S$, M = 235.34, triclinic, space group P-1, at 296 K: a = 7.1274(3), b = 11.6764(6), c = 14.5849(7) Å, α = 98.754(2), β = 100.214(2), γ = 97.009(2)°, V = 1166.70(10) Å^3 , Z = 4, d_{calc} = 1.340 g·cm^{-3} , μ = 0.254 mm^{-1} , a total of 37381 (θ_{max} = 29.16°), 6287 unique (R_{int} = 0.0363), 4869 [I > 2σ(I)], 305 parameters. GooF = 0.983, R_1 = 0.0364, wR_2 = 0.0962 [I > 2σ(I)], R_1 = 0.0532, wR_2 = 0.1096 (all data), max/min diff. peak 0.33/-0.28 e·Å^{-3}.

Crystal data **15b**: $C_{22}H_{31}N_3OS$, M = 385.56, monoclinic, space group $P2_1$, at 296 K: a = 7.1954(9), b = 22.193(3), c = 13.9687(19) Å, β = 104.282(5)° , V = 2161.7(5) Å^3 , Z = 4, d_{calc} = 1.185 g·cm^{-3} , μ = 0.166 mm^{-1} , a total of 43798 (θ_{max} = 27.25°), 9632 unique (R_{int} = 0.0459), 8032 [I > 2σ(I)], 486 parameters. GooF = 1.044, R_1 = 0.0525, wR_2 = 0.1489 [I > 2σ(I)], R_1 = 0.0686, wR_2 = 0.1733 (all data), max/min diff. peak 0.43/-0.26 e·Å^{-3}.

Table S1. The combination index (CI) values for different concentrations of topotecan and Tdp1 inhibitors.

CI Data for Non-Constant Combo: (Tpc+20 μM **14a**)

**Dose Tpc, μM**	**Dose 14a, μM**	**Effect**	**CI**
0.3	20.0	0.17	NaN
0.6	20.0	0.47	NaN
1.2	20.0	0.81	0.28719
2.5	20.0	0.92	0.44610
5.0	20.0	0.96	0.71769

CI Data for Non-Constant Combo: (Tpc + 20 μM **14b**)

**Dose Tpc, μM**	**Dose 14b, μM**	**Effect**	**CI**
0.3	20.0	0.94	0.00419
0.5	20.0	0.97	0.00165
1.0	20.0	0.98	0.00117
2.0	20.0	0.98	0.00166
4.0	20.0	0.99	0.00113

CI Data for Non-Constant Combo: (Tpc + 50 μM **15a**)

**Dose Tpc, μM**	**Dose 15a, μM**	**Effect**	**CI**
0.3	50.0	0.27	NaN
0.5	50.0	0.53	0.07887
1.0	50.0	0.73	0.06365
2.0	50.0	0.83	0.06893
4.0	50.0	0.9	0.07307

CI Data for Non-Constant Combo: (Tpc + 50 μM **15b**)

**Dose Tpc, μM**	**Dose 15b, μM**	**Effect**	**CI**
0.3	50.0	0.48	NaN
0.5	50.0	0.69	0.05563
1.0	50.0	0.84	0.04653
2.0	50.0	0.9	0.05382
4.0	50.0	0.91	0.09564

CI Data for Non-Constant Combo: (Tpc+20 μM **16**)

**Dose Tpc, μM**	**Dose 16, μM**	**Effect**	**CI**
0.3	20.0	0.035	NaN
0.6	20.0	0.1	NaN
1.2	20.0	0.36	NaN
2.5	20.0	0.75	0.51140
5.0	20.0	0.85	0.87206

CI Data for Non-Constant Combo: (Tpc+20 μM **17**)

**Dose Tpc, μM**	**Dose 17, μM**	**Effect**	**CI**
0.3	20.0	1.0E-4	NaN
0.6	20.0	0.02	NaN
1.2	20.0	0.25	NaN
2.5	20.0	0.6	0.79239
5.0	20.0	0.81	1.17556

Figure S2. The cytotoxicity of the monoterpene derivatives against HeLa and HEK293A cells, as measured by the EZ4U test. Error bars show standard deviations.

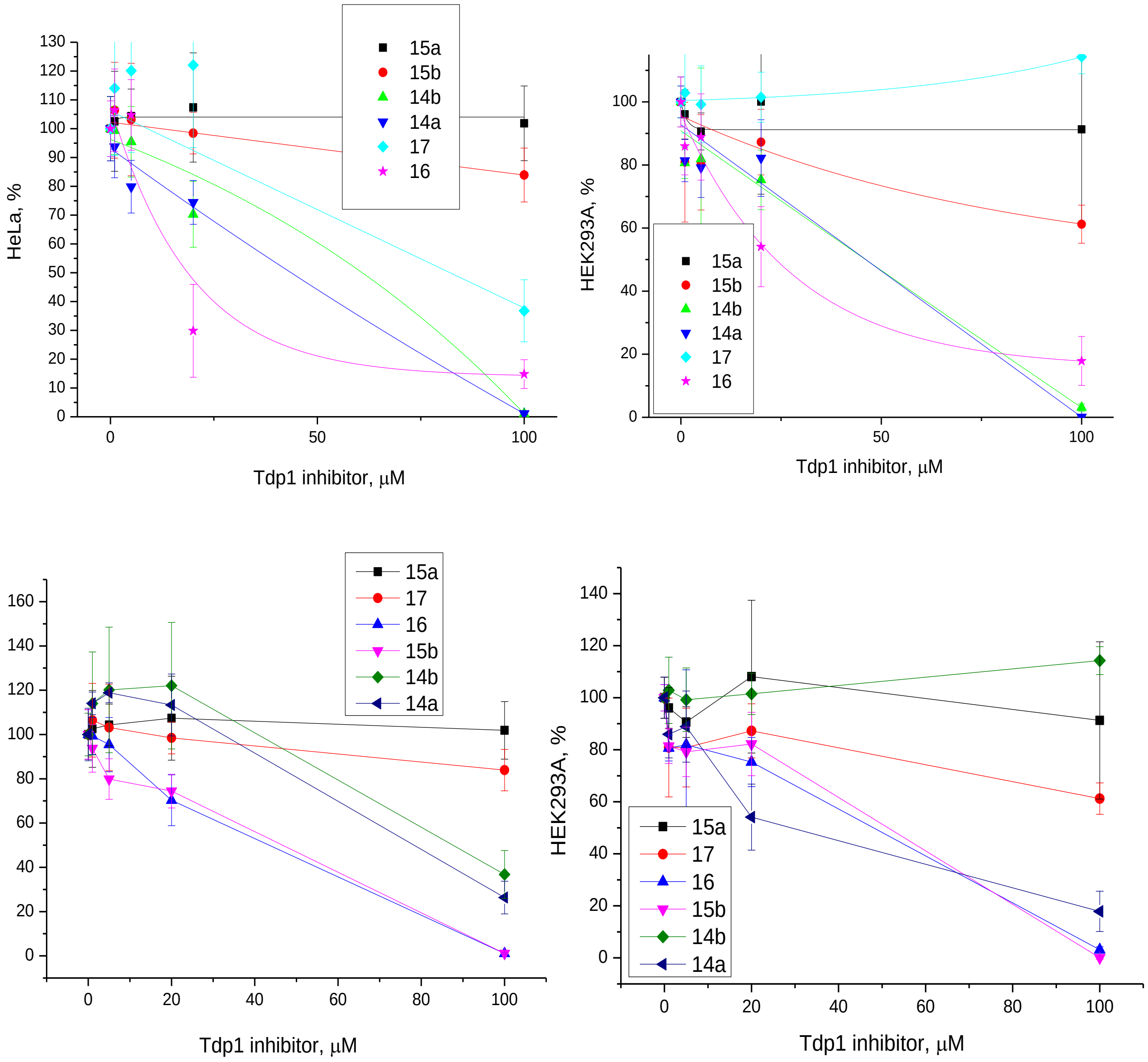

Figure S3. The influence of the TDP1 inhibitors on topotecan cytotoxicity derivatives against HeLa and HEK293A cells. Error bars show standard deviations.

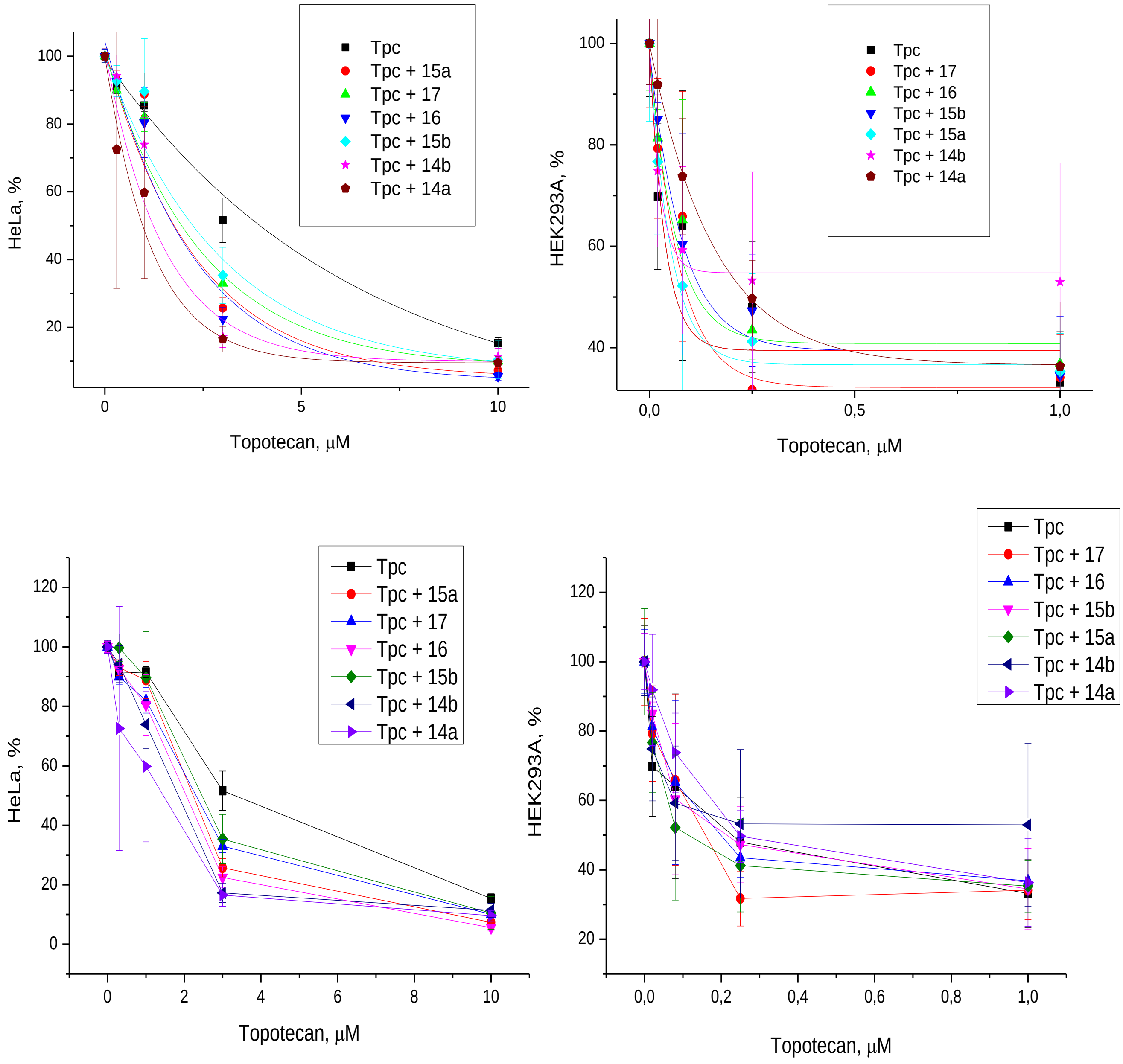

Figure S4. The influence of topotecan on the adamantane-monoterpenoid derivatives' cytotoxicity.

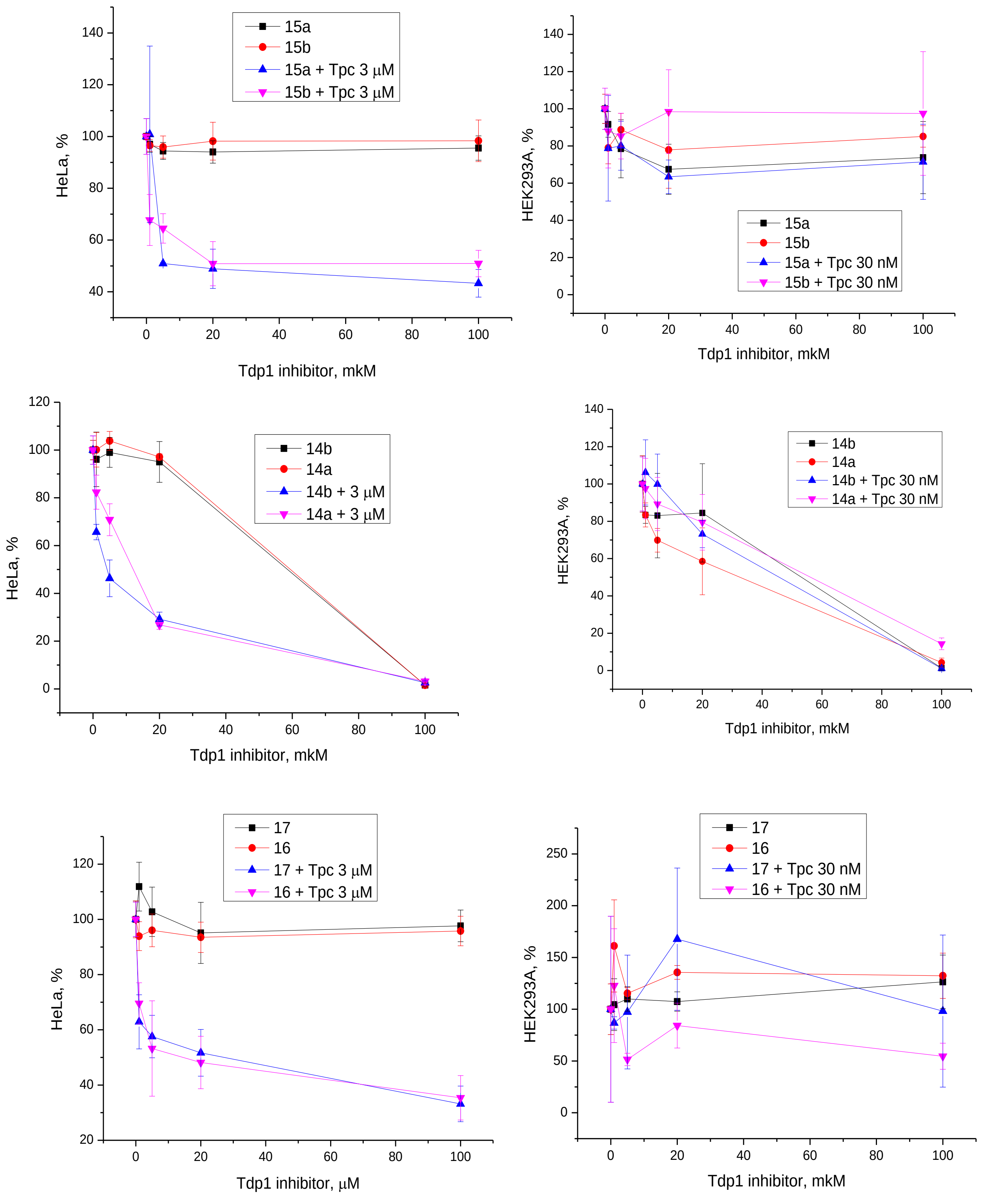

Table S2. The binding affinities as predicted by the scoring functions used to the catalytic Tdp1 binding pocket.

Ligands	ASP	ChemPLP	CS	GS	IC_{50} (μM)
**14a**	30.7	49.9	24.8	57.7	5.5
**14b**	29.3	53.9	26.3	57.3	6.1
**15a**	30.2	52.2	24.7	52.9	6.1
**15b**	30.2	51.9	23.2	53.0	5.6
**16**	31.1	52.2	25.6	52.3	34
**17**	29.7	51.2	24.3	55.9	>30

Table S3. The molecular descriptors and their corresponding Known Drug Indexes 2a and 2b ($KDI_{2a/2b}$). The R^2 numbers derived from correlation with the Tdp1 IC_{50} values; the Tdp1 IC_{50} value of **17** is set to 30 μM.

	RB	MW	HD	HA	Log P	PSA	KDI_{2A}	KDI_{2B}
**14a**	4	371.6	1	2.5	6.1	42.3	4.53	0.12
**14b**	4	371.6	1	2.5	6.0	41.6	4.53	0.12
**15a**	3	385.6	1	4.5	5.0	61.0	5.27	0.43
**15b**	3	385.6	1	4.5	5.0	59.5	5.25	0.42
**16**	2	399.6	0	5.5	4.1	81.9	5.15	0.35
**17**	4	449.6	1	8.5	3.2	96.5	5.21	0.40
**Tdp1 R^2:**	0.150(-)	0.576(+)	0.493(-)	0.590(+)	0.710(-)	0.772(+)	0.165(+)	0.123(+)

Table S4. Definition of lead-like, drug-like and Known Drug Space (KDS) in terms of molecular descriptors. The values given are the maxima for each descriptor for the volumes of chemical space used.

	**Lead-like Space**	**Drug-like Space**	**Known Drug Space**
Molecular weight (g mol^{-1})	300	500	800
Lipophilicity (Log P)	3	5	6.5
Hydrogen bond donors (HD)	3	5	7
Hydrogen bond acceptors (HA)	3	10	15
Polar surface area (Å^2) (PSA)	60	140	180
Rotatable bonds (RB)	3	10	17

^{1}H, ^{13}C NMR & DFS spectra

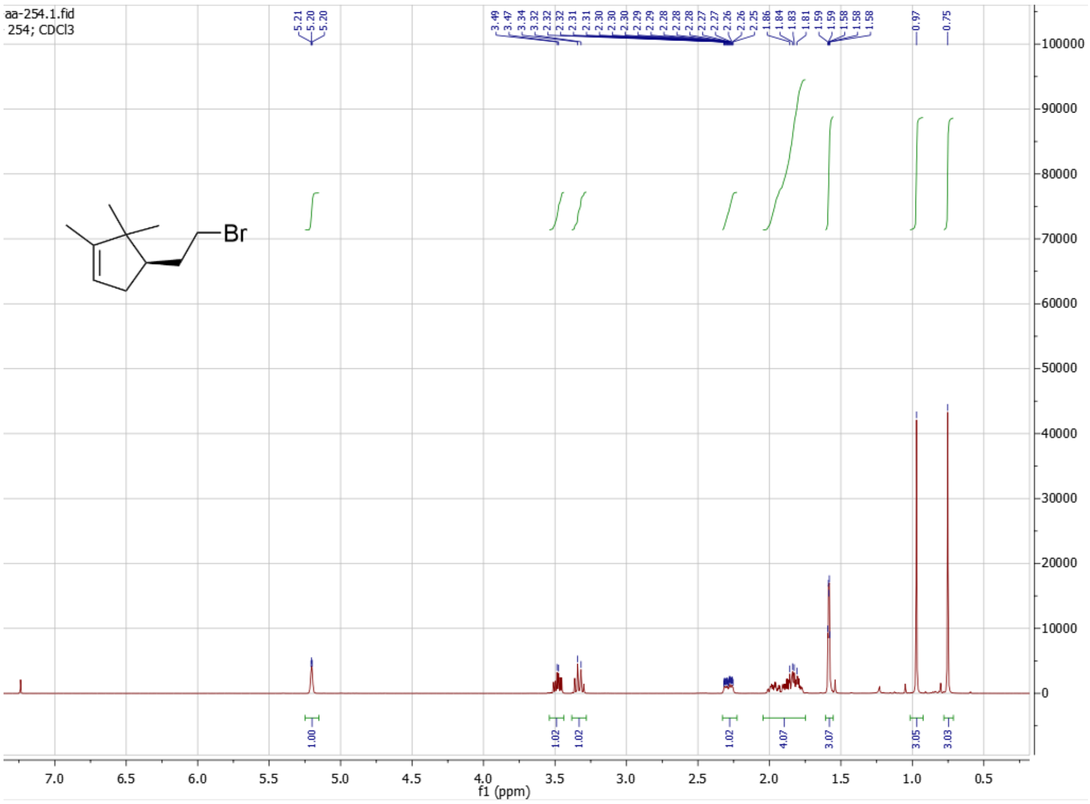

Figure S5. The ^{1}H NMR spectrum of **8.**

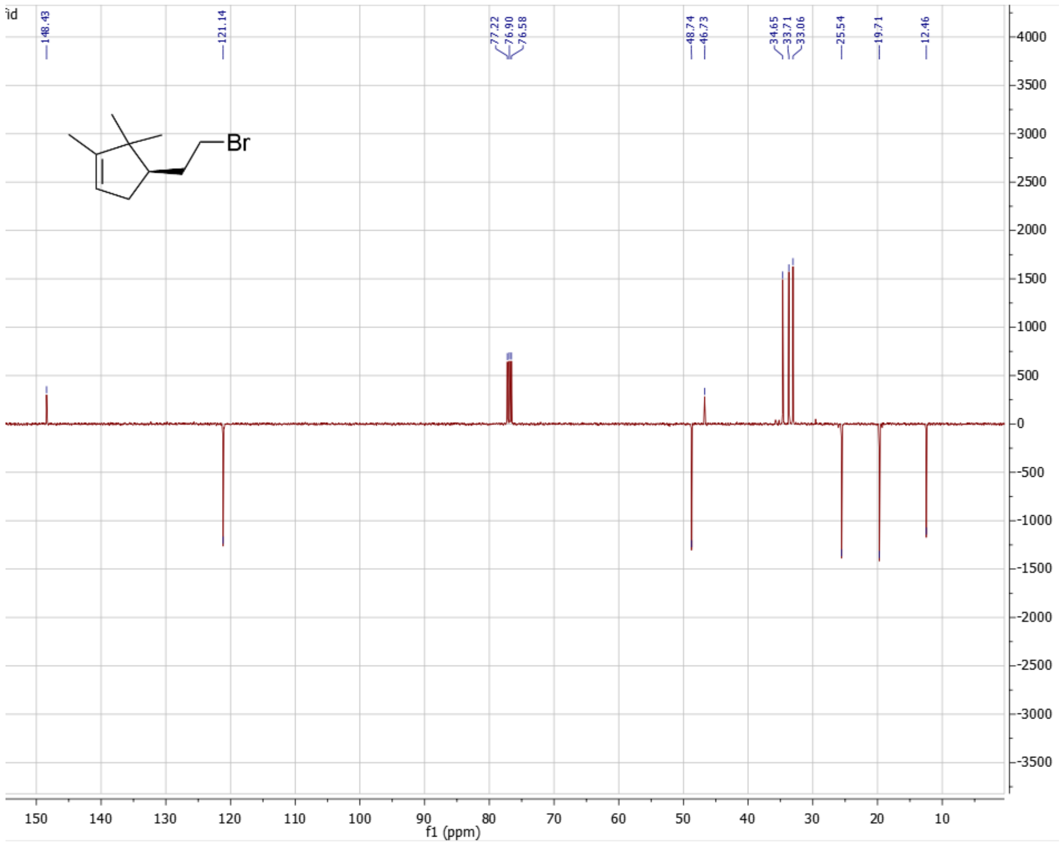

Figure S6. The ^{13}C NMR spectrum of **8.**

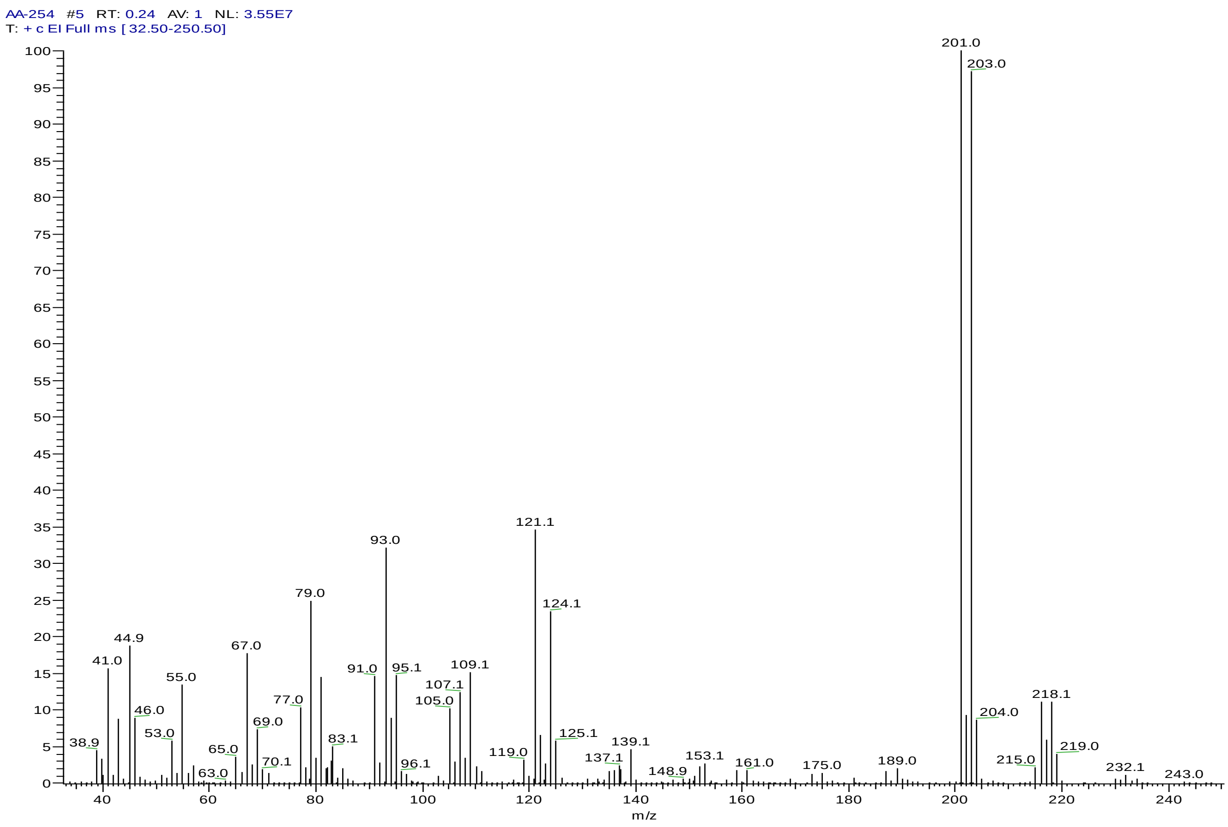

Figure S7. The DFS spectrum of **8.**

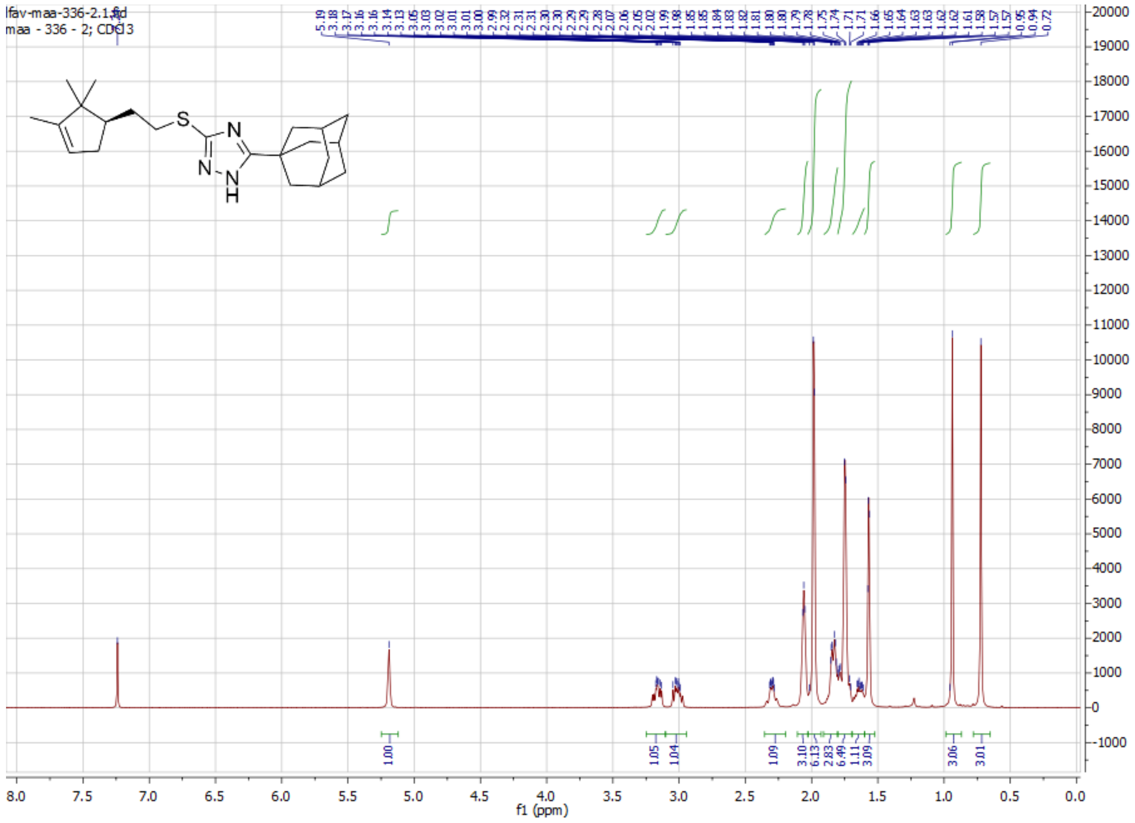

Figure S8. The ^{1}H NMR spectrum of **14a.**

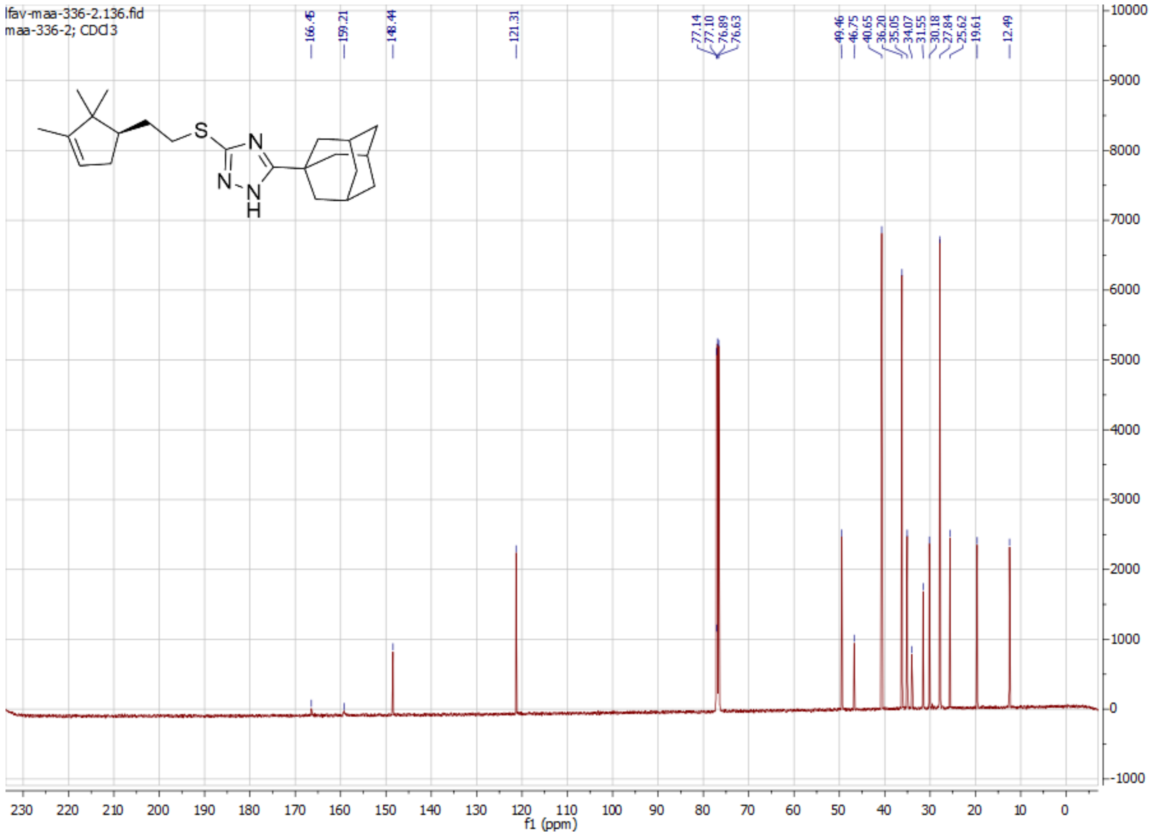

Figure S9. The ^{13}C NMR spectrum of **14a.**

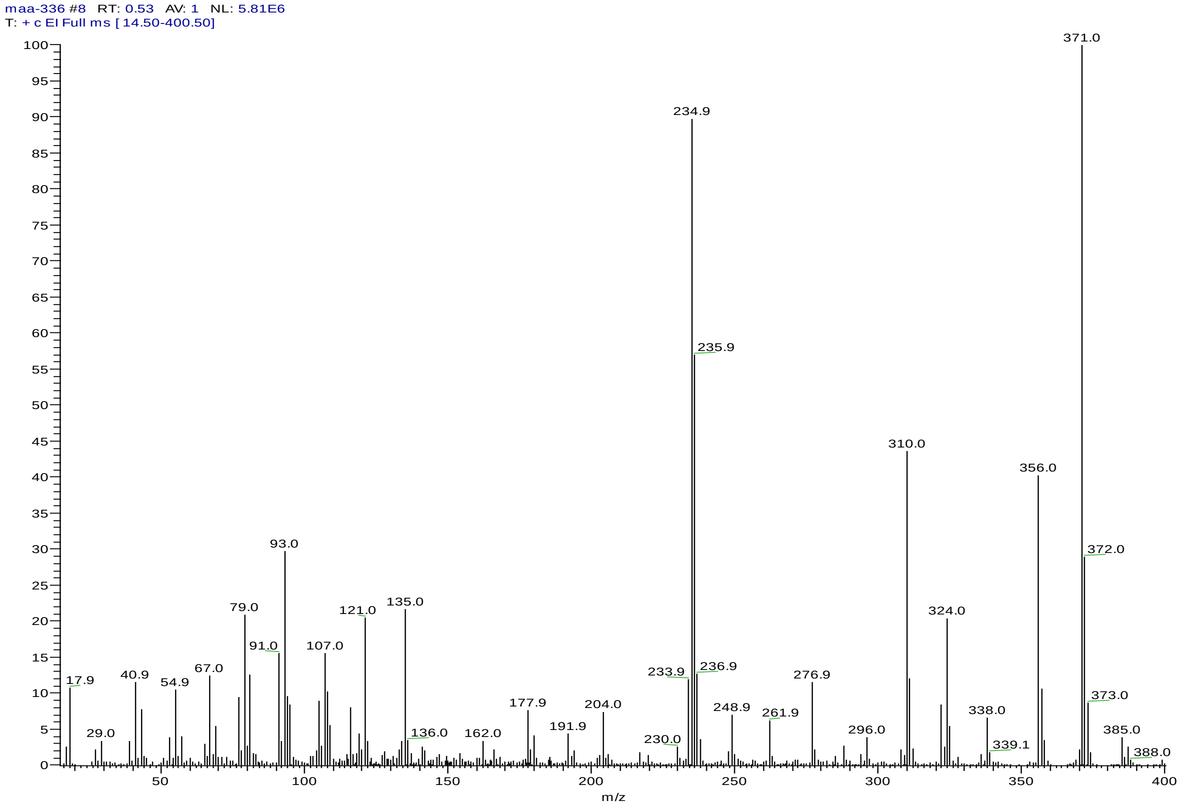

Figure S10. The DFS spectrum of **14a.**

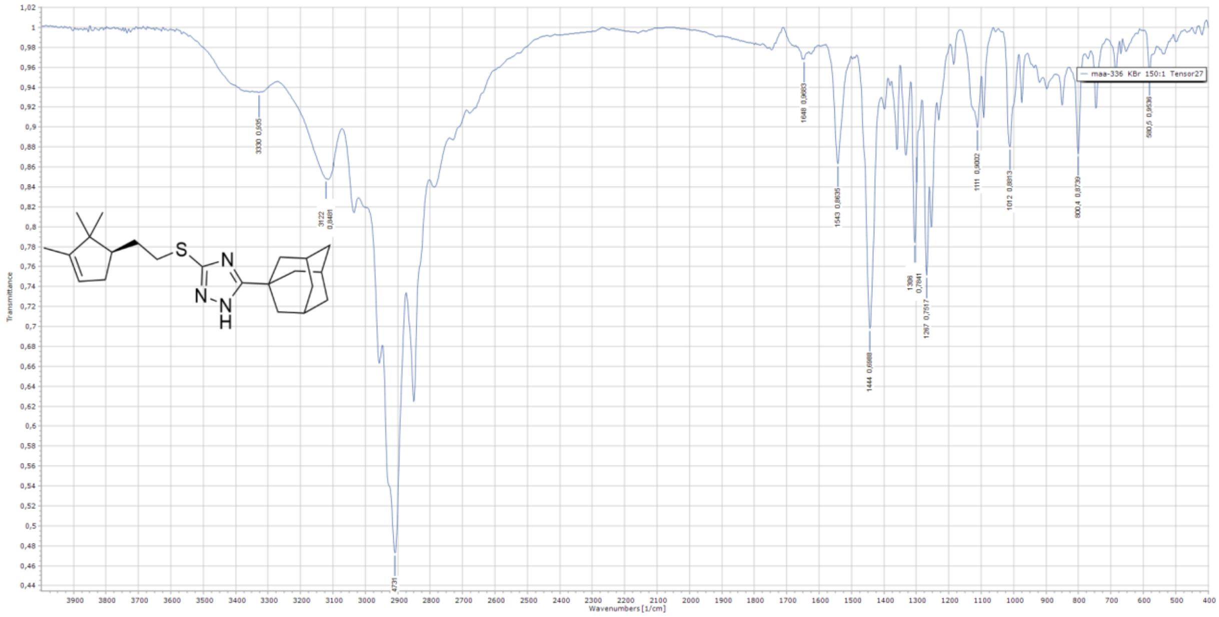

Figure S11. The IR spectrum of **14a**.

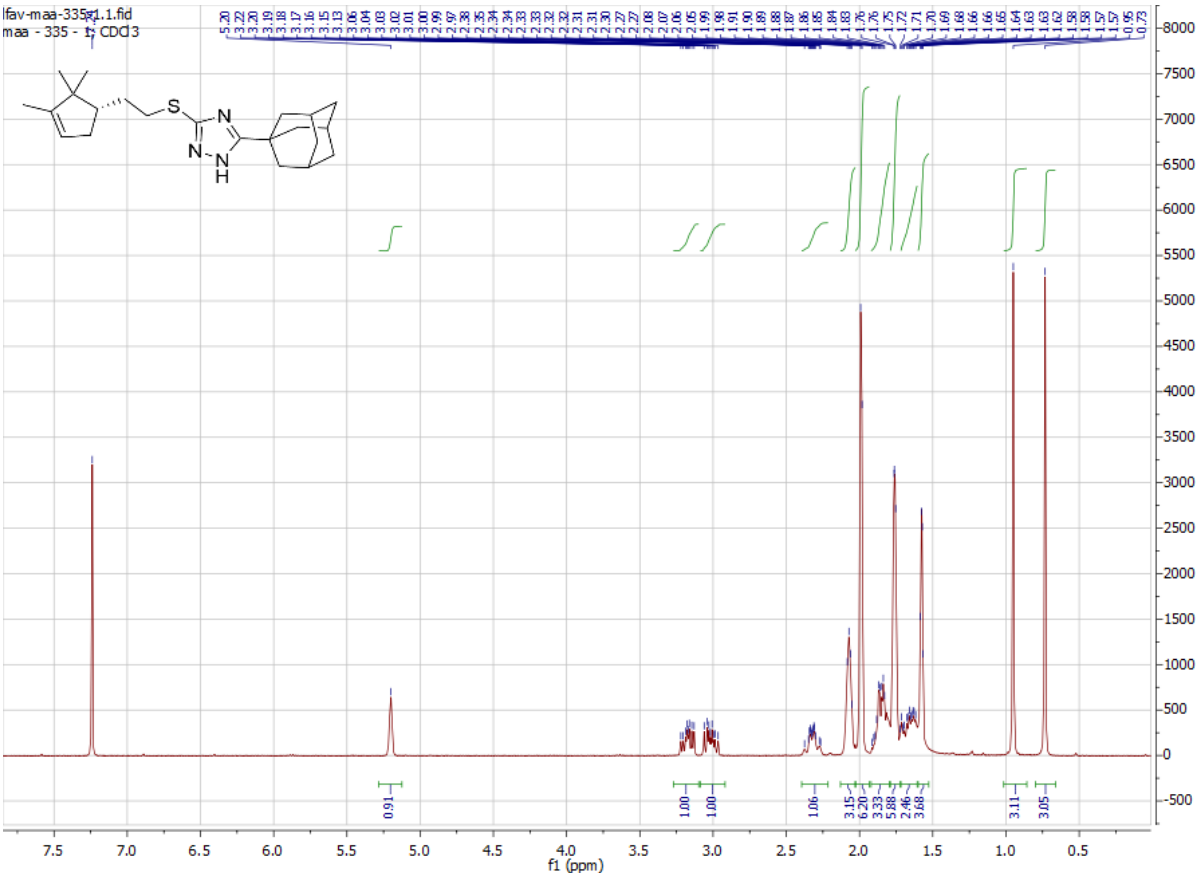

Figure S12. The ^{1}H NMR spectrum of **14b.**

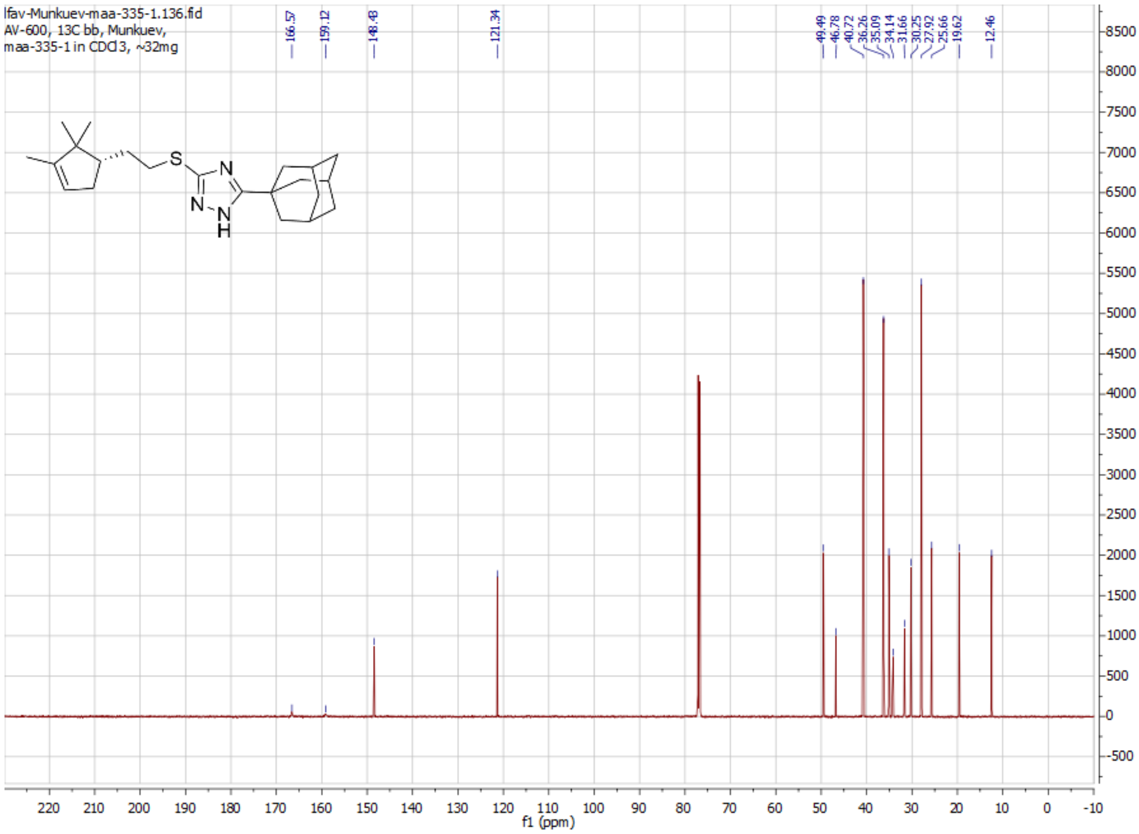

Figure S13. The ^{13}C NMR spectrum of **14b.**

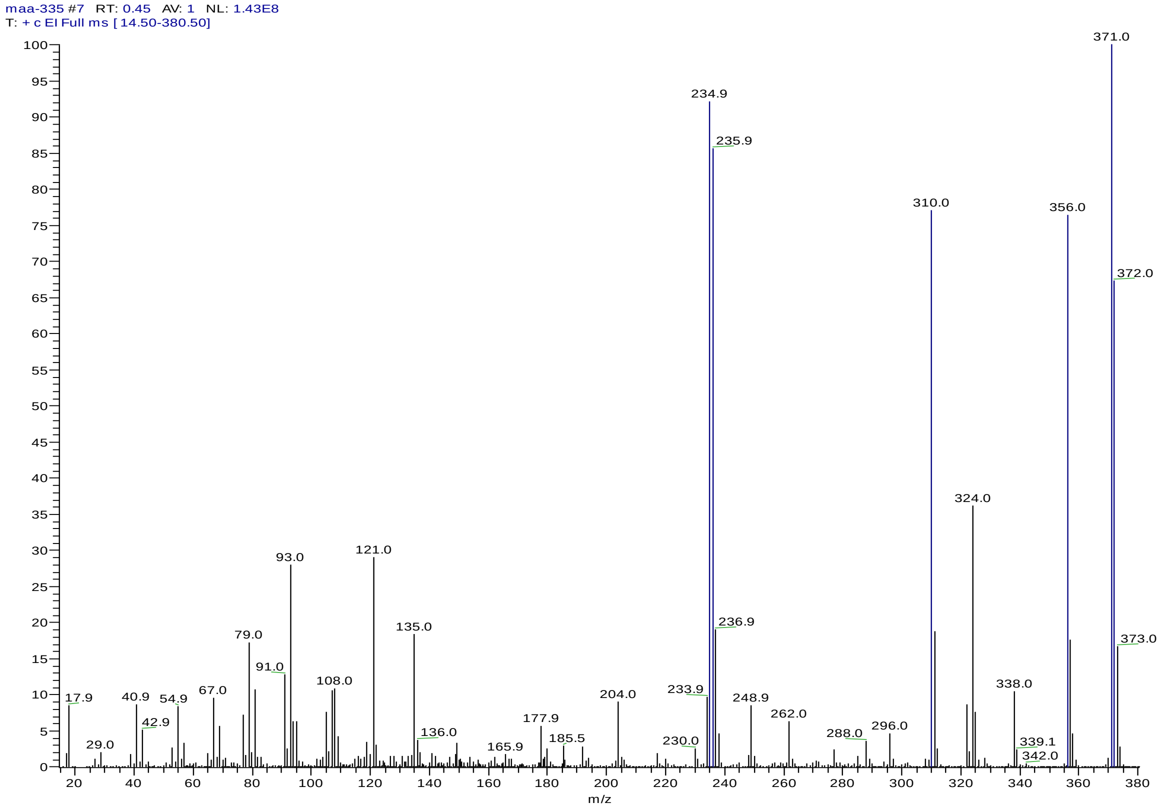

Figure S14. The DFS spectrum of **14b.**

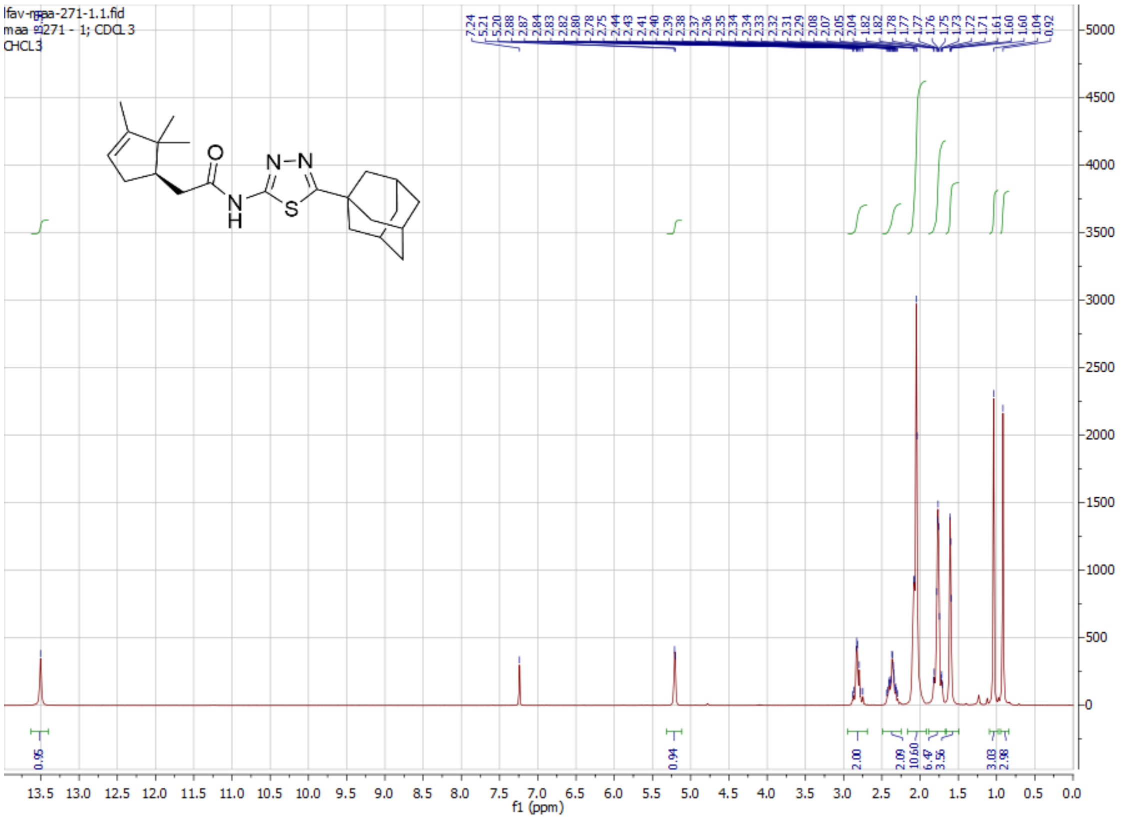

Figure S15. The ^{1}H NMR spectrum of **15a.**

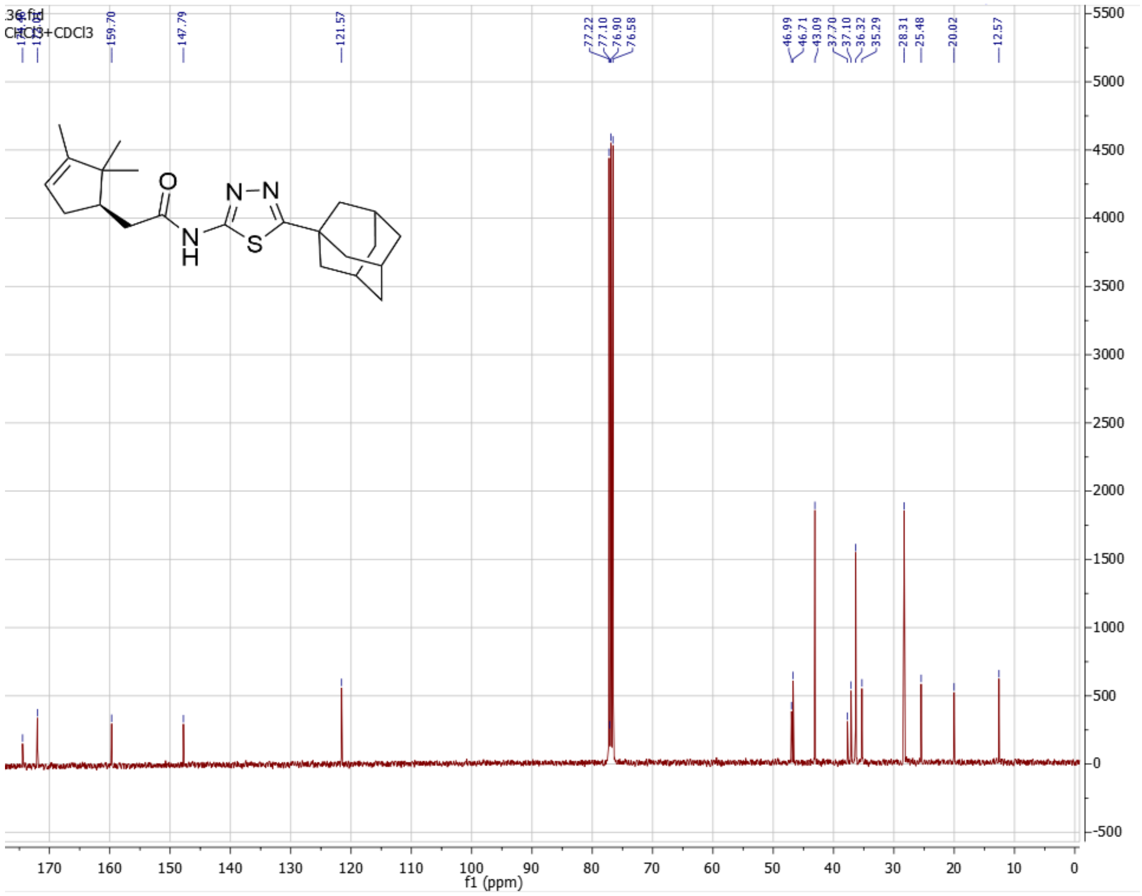

Figure S16. The ^{13}C NMR spectrum of **15a.**

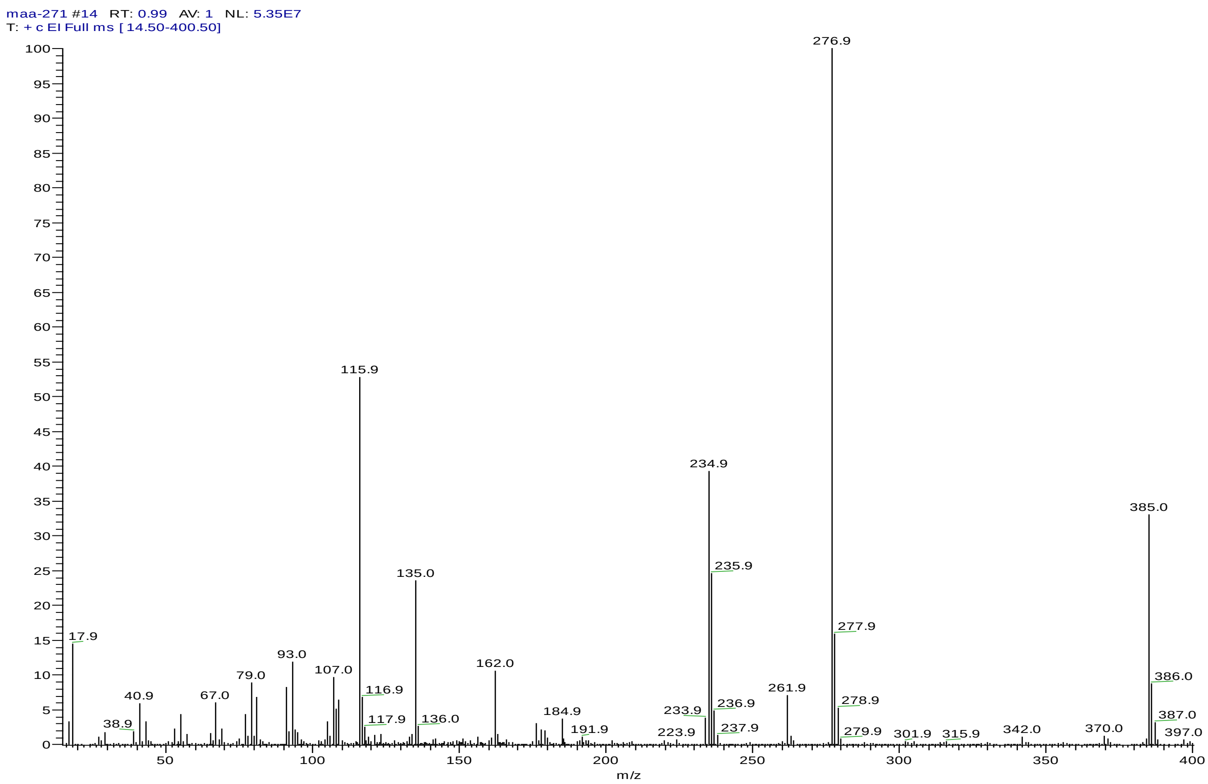

Figure S17. The DFS spectrum of **15a.**

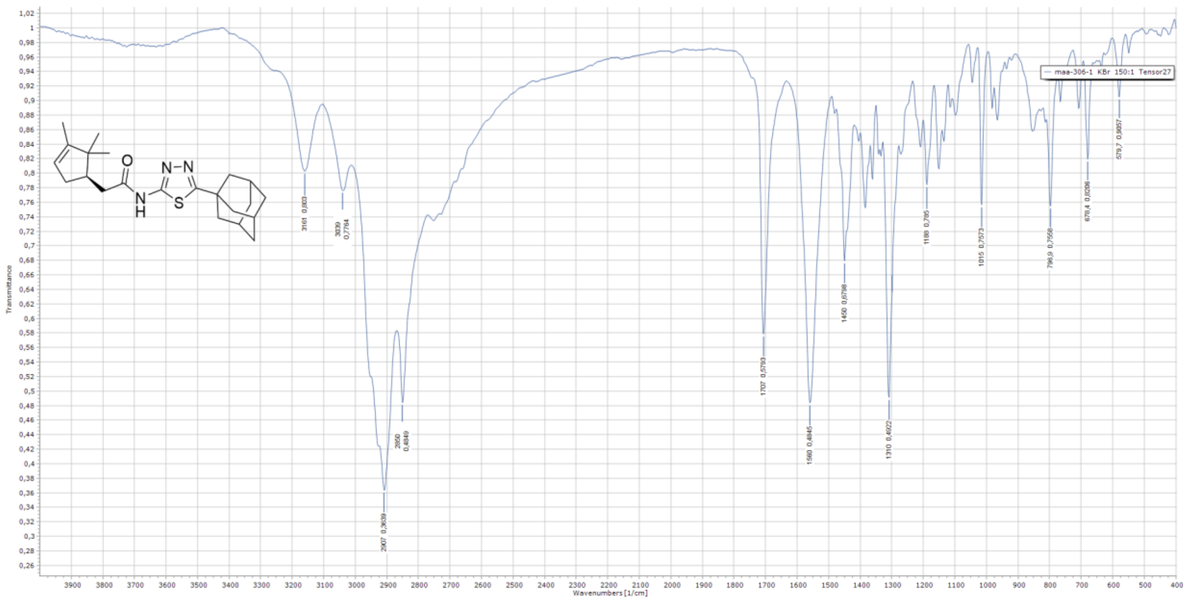

Figure S18. The IR spectrum of **15a**.

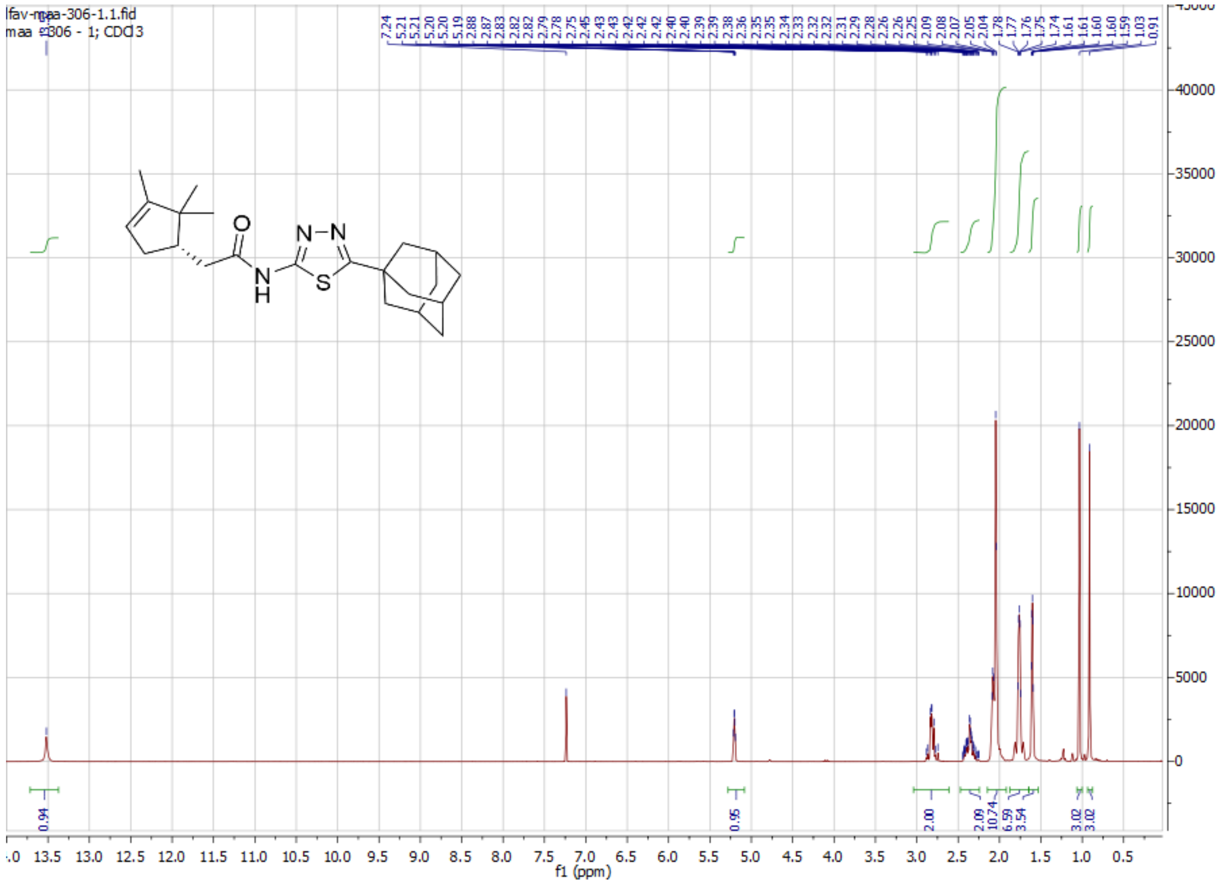

Figure S19. The ^{1}H NMR spectrum of **15b.**

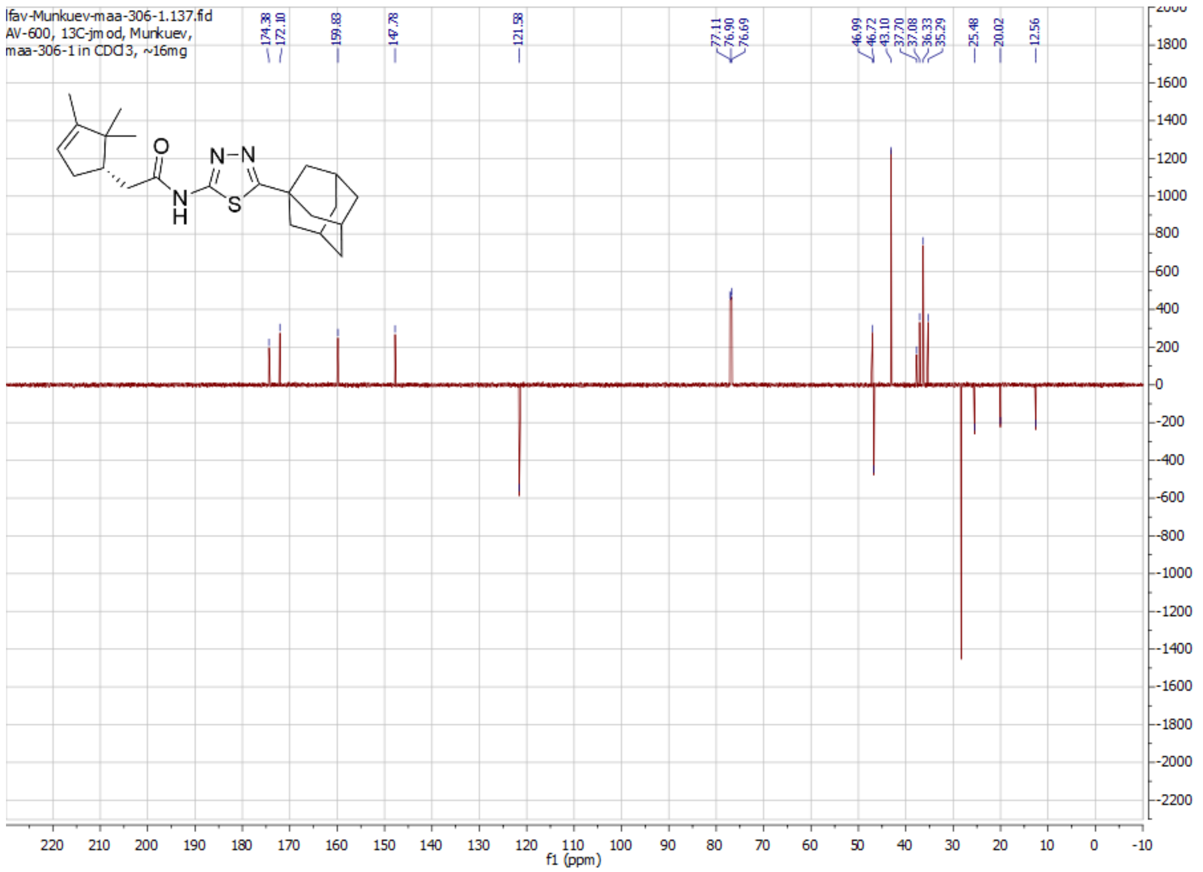

Figure S20. The ^{13}C NMR spectrum of **15b.**

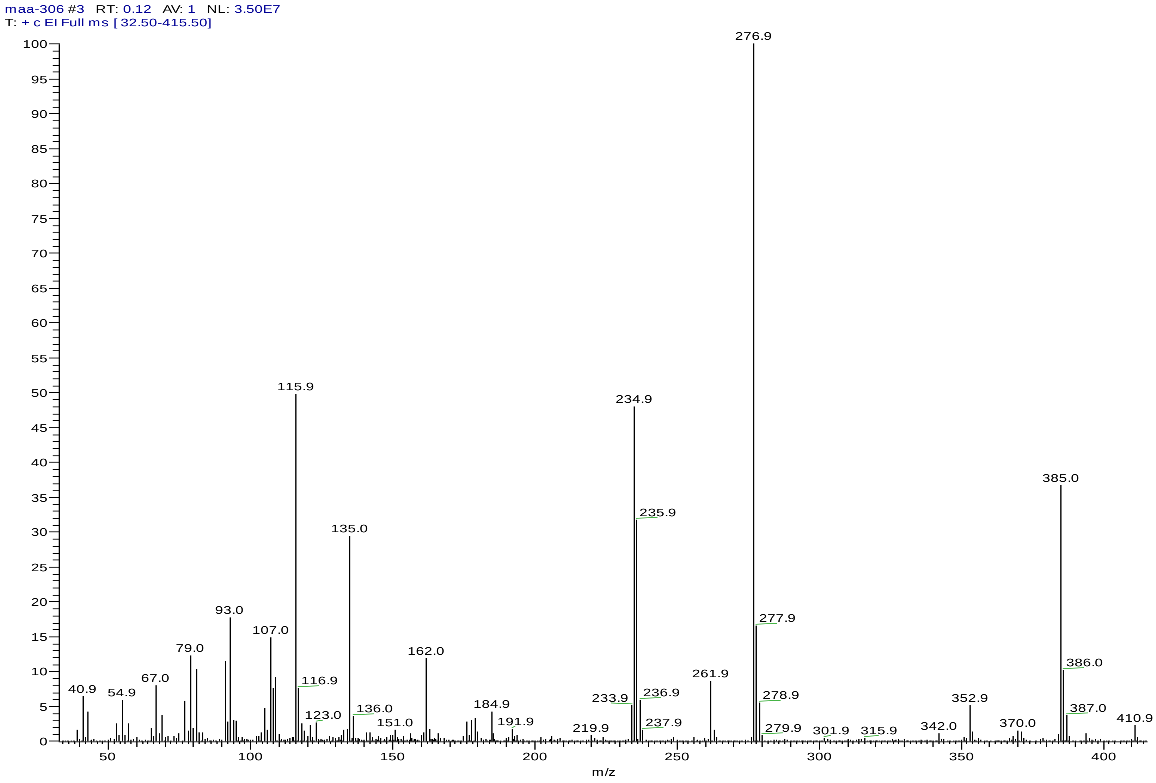

Figure S21. The DFS spectrum of **15b.**

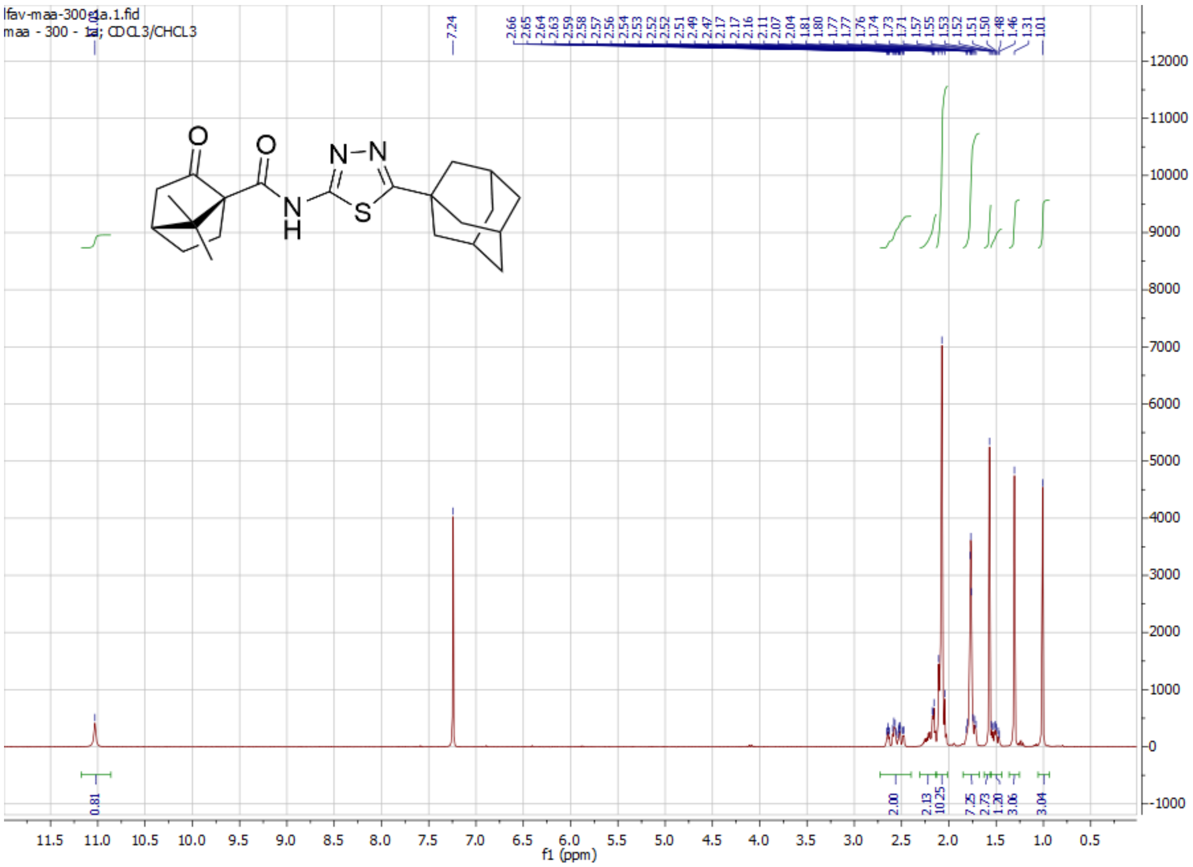

Figure S22. The ^{1}H NMR spectrum of **16.**

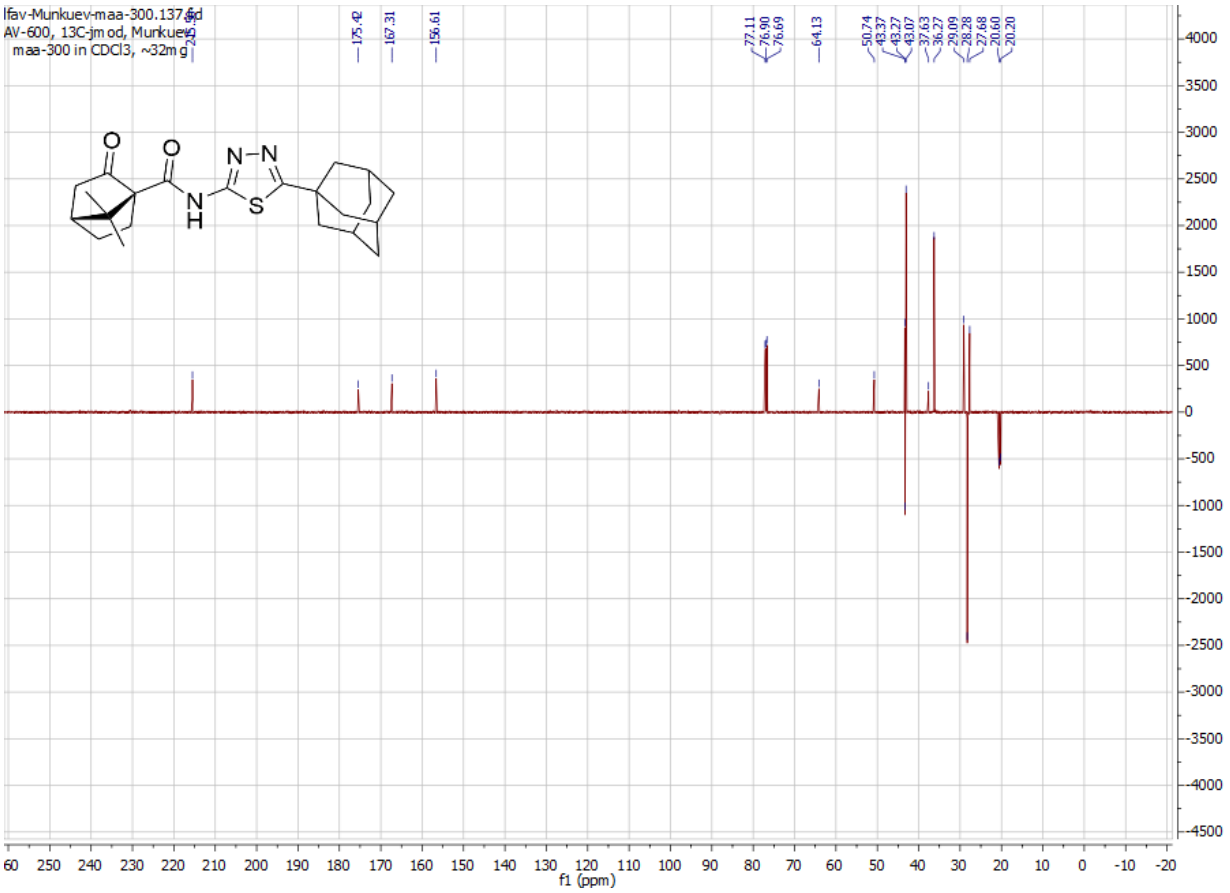

Figure S23. The ^{13}C NMR spectrum of **16.**

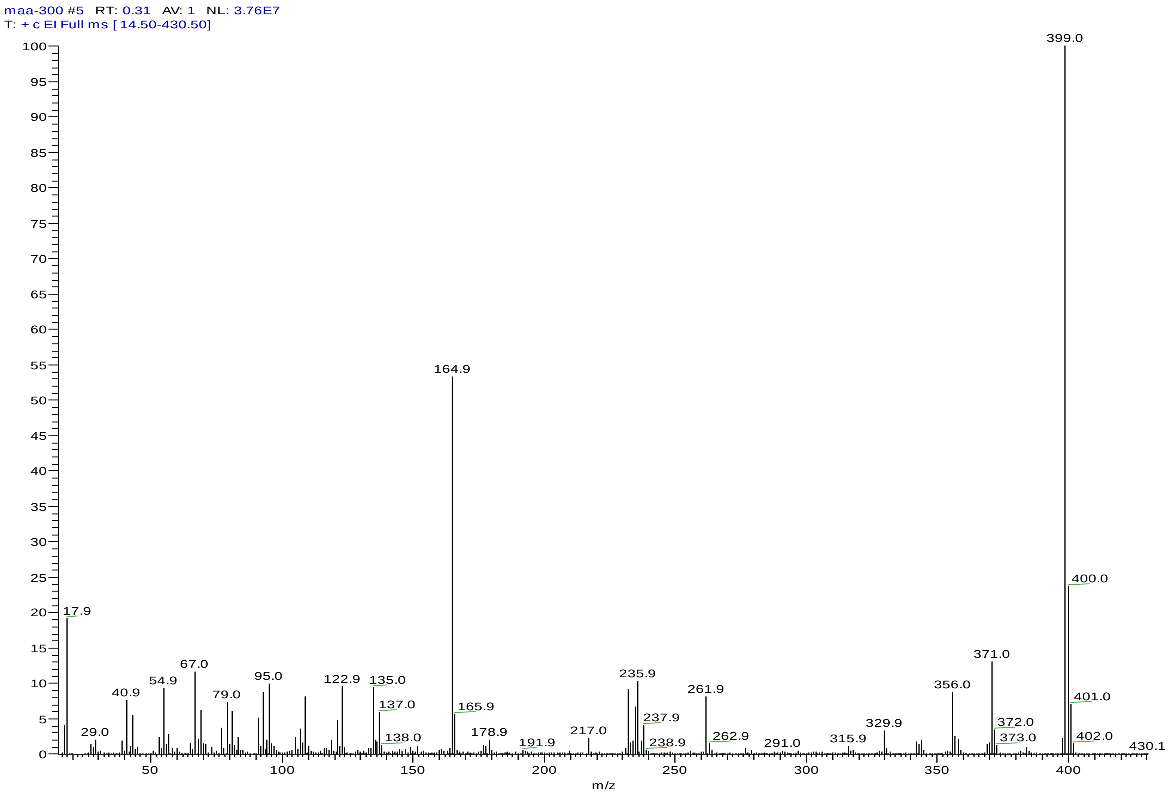

Figure S24. The DFS spectrum of **16.**

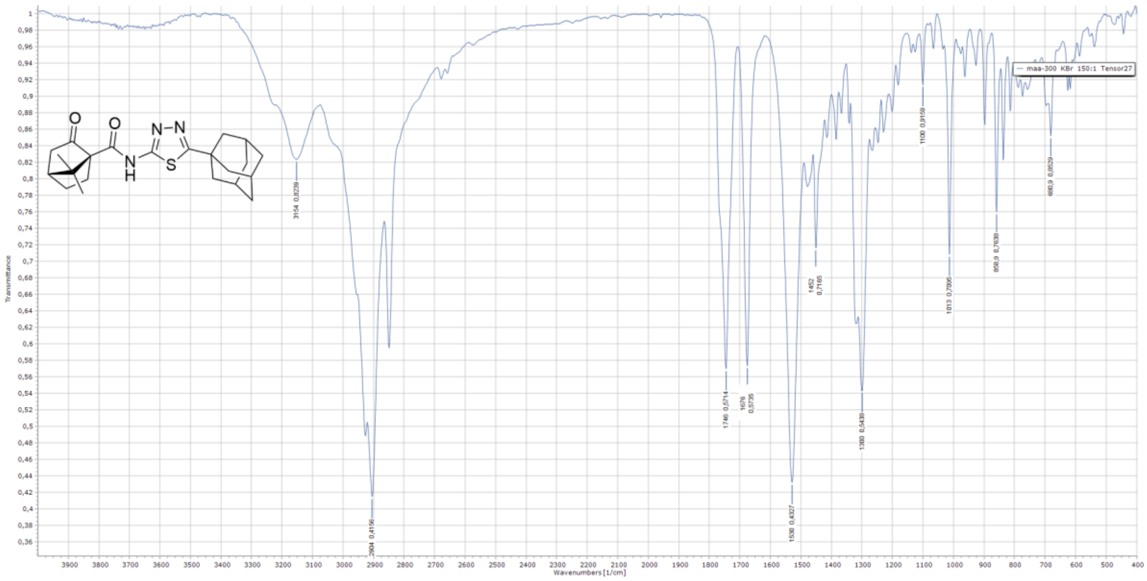

Figure S25. The IR spectrum of **16**.

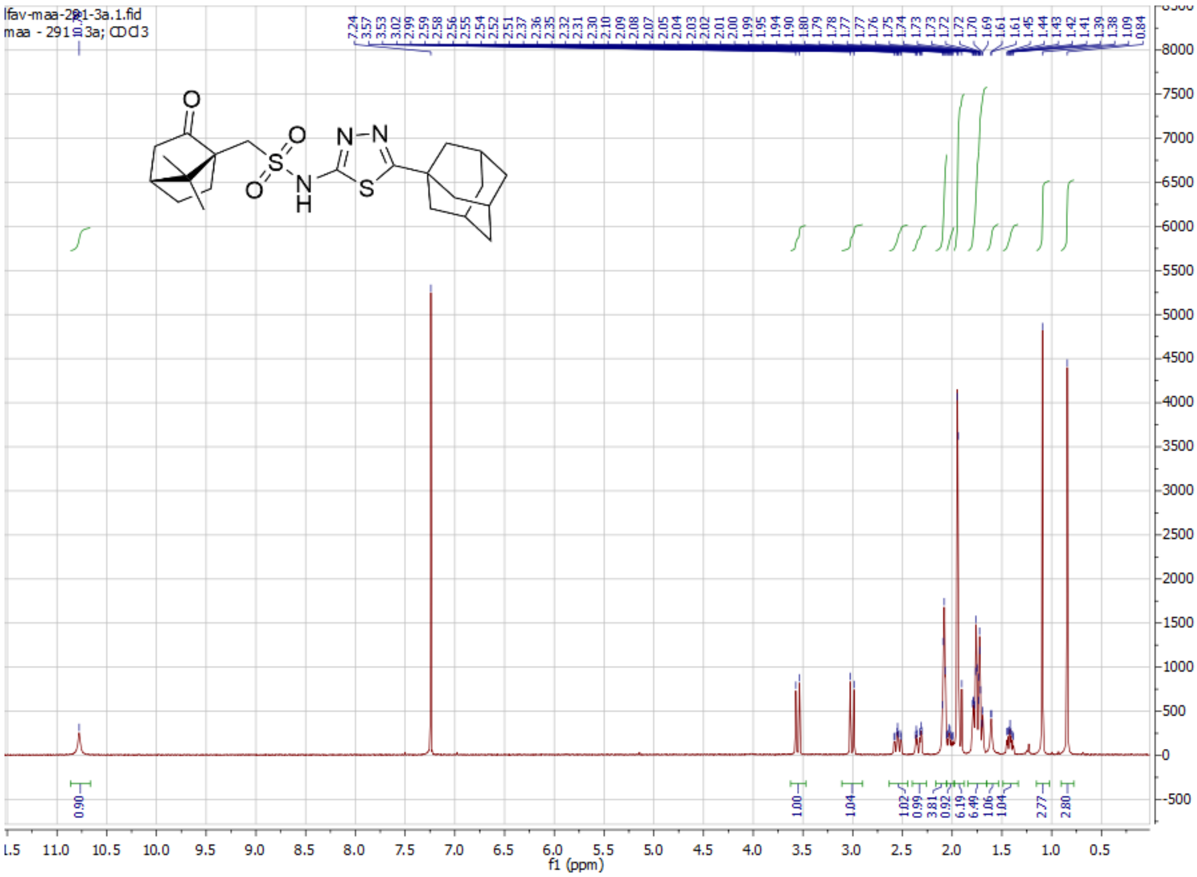

Figure S26. The ^{1}H NMR spectrum of **17.**

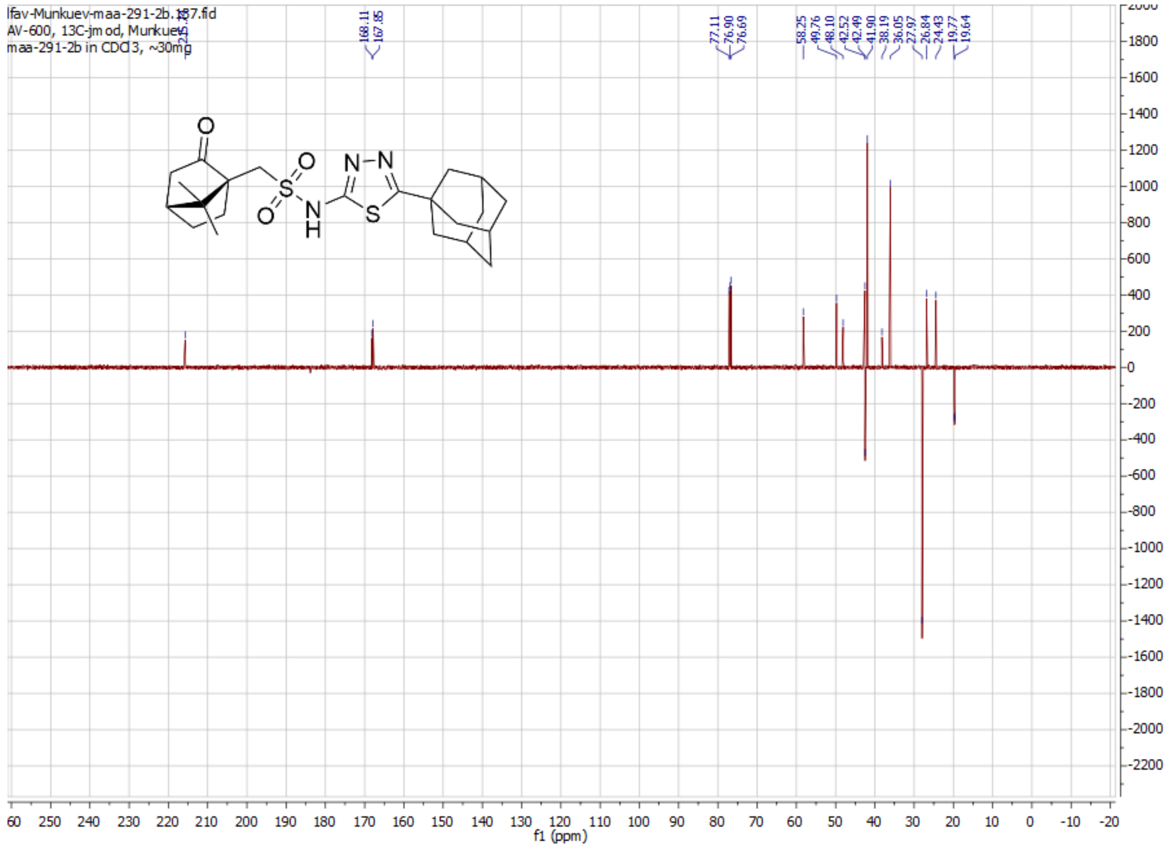

Figure S27. The ^{13}C NMR spectrum of **17**.

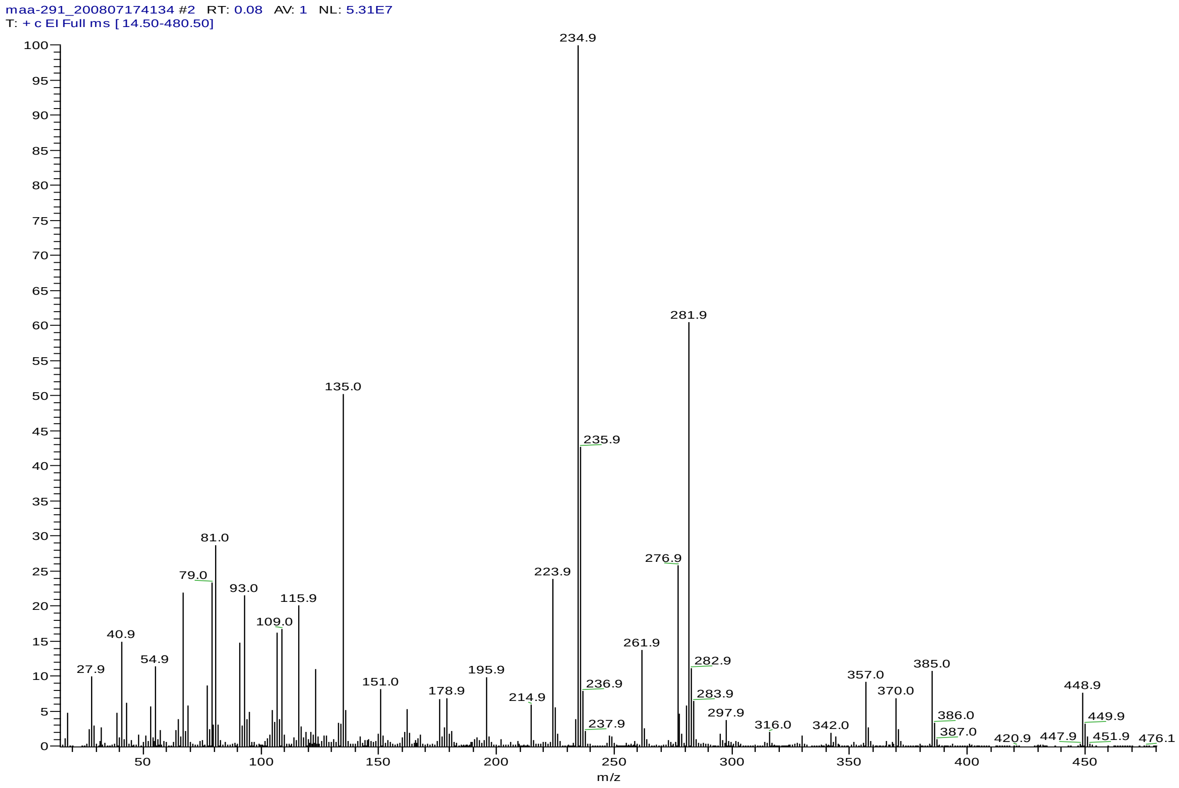

Figure S28. The DFS spectrum of **17**.

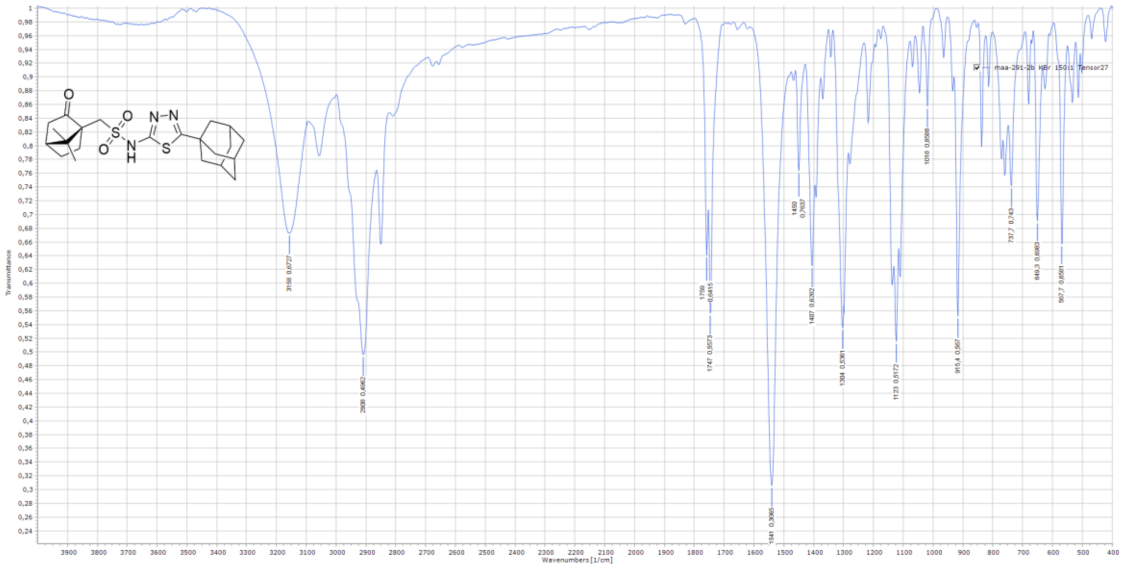

Figure S29. The IR spectrum of **17**.
